# Novel transcriptomic alterations in poorly differentiated endometrial carcinomas: evidence from South African women

**DOI:** 10.3389/fonc.2026.1694662

**Published:** 2026-03-24

**Authors:** Thulo Molefi, Mohammed Alaouna, Talent Chipiti, Hannah Sebitloane, Zodwa Dlamini

**Affiliations:** 1 Discipline of Obstetrics and Gynaecology, School of Clinical Medicine, University of KwaZulu-Natal, Durban, South Africa; 2 SAMRC Precision Oncology Research Unit (PORU), DSI/NRF SARChI Chair in Precision Oncology and Cancer Prevention (POCP), Pan African Research Institute (PACRI), University of Pretoria, Pretoria, South Africa; 3 Department of Medical Oncology, University of Pretoria, Pretoria, South Africa; 4 Wolfson Wohl Cancer Research Centre, School of Cancer Sciences, University of Glasgow, Glasgow, United Kingdom

**Keywords:** alternative splicing, differential gene expression, endometrial cancer, molecular biomarker identification, novel transcript discovery, population-specific transcriptomics, RNA-seq FFPE, zinc-finger transcription factors

## Abstract

**Background:**

Poorly differentiated endometrial carcinoma in Black African women is under-characterized at the transcriptomic level, although it is known for aggressive subtypes. We conducted the first RNA-seq analysis of formalin-fixed, paraffin-embedded (FFPE) tumors from Black South African women to explore population-specific gene expression, alternative splicing, and novel isoforms.

**Methods:**

Seventy-six FFPE tumor samples passed RNA quality thresholds (DV200 ≥ 30%) and were sequenced on the PacBio Onso™ short-read platform. StringTie assembled transcripts and estimated transcripts per million (TPMs); differential expression was quantified by mean log_2_ fold-change (|log_2_FC| ≥ 0.5). SUPPA2 computed percent spliced-in (PSI) values for five splicing event classes. Reactome over-representation analysis identified enriched pathways, and novel isoforms were validated by read coverage and expression support.

**Results:**

Cohort-level analysis (n = 76) revealed the coordinated activation of signaling and broad repression of transcriptional programs. We detected 17,990 dysregulated transcripts (4,483 upregulated and 13,507 downregulated; range −4.81 to +2.99). Prominent upregulated transcripts included GJA1 (+2.99), GRN (+2.60), ZC3H3 (+2.58), PCSK4 (+2.55), HSPB7 (+2.55), BICRA (+2.49), LINC02692 (+2.23), MKNK2 (+2.03), and RARA (+0.76). Strong downregulation was observed for MRPL15 (−4.81), ZSCAN23 (−4.46), NUF2 (−4.37), RTN1 (−4.33), EIF3I (−4.31), CDC73 (−3.36), MYC (−2.96), FRS2 (−2.90), and zinc-finger factors including ZNF569 (−3.36), ZNF573 (−2.92), ZNF793 (−2.33), and ZNF382 (−1.87). Reactome enrichment linked the upregulated set to MAPK family/GPCR signaling, while the downregulated categories were associated with gene expression (transcription), RNA polymerase II transcription, cell cycle, and metabolism. Alternative splicing was pervasive, with recurrent high-magnitude events at cancer-relevant loci including GJA1 (SE), NF2 (A3/A5), LIN37 (A5), ECT2 (A3/A5), BCL2L11 (A3), UQCRH (A5), CSE1L (A3), BROX (A3), and multiple ZNFs. StringTie identified previously unannotated isoforms at LINC02692 (+2.23), LINC01605 (−0.62), ZNF793 (−2.33), ZNF382 (−1.87), and retained-intron transcripts at RAD51 (+1.30), RPS24 (−0.97), and RPS3A (−0.62).

**Conclusions:**

Poorly differentiated endometrial carcinomas in Black South African women show a distinct MAPK-linked activation pattern, along with transcriptional repression and extensive splicing changes. Aligning with findings in African Americans, this cohort highlights the unique aspects of isoform-resolved splicing and zinc-finger repression, suggesting that translational control, retinoid signaling, and RNA processing may be targets for biomarkers and therapy.

## 1 Introduction

Endometrial carcinoma is the most prevalent form of gynecological cancer in developed nations, with a global increase in incidence, primarily driven by demographic transitions, rising obesity rates, and shifts in reproductive health paradigms (1, 2). Aggressive subtypes of endometrial cancer, particularly uterine serous carcinoma and carcinosarcoma, pose significant treatment challenges owing to late-stage diagnoses, rapid disease progression, elevated recurrence rates, and contrasting mortality outcomes, despite representing a smaller segment of overall cases (3–5). Extensive research, notably through initiatives like The Cancer Genome Atlas (TCGA), has made significant strides in elucidating the genetic landscape of these aggressive tumor types by identifying pivotal mutations, such as in the TP53 gene, and key alterations in molecular pathways like PI3K/AKT/mTOR (6, 7).

Studies have indicated that endometrial tumors in women of African descent often exhibit distinct genetic and clinical profiles, including a predominance of the TCGA-defined copy-number-high (serous-like) molecular subtype, which is characterized by extensive somatic copy number alterations, widespread chromosomal instability, and high levels of aneuploidy, together with increased frequencies of TP53 mutations and oncogenic amplifications such as Erb-B2 Receptor Tyrosine Kinase 2 (ERBB2), also known as Human Epidermal Growth Factor Receptor 2 (HER2) (8, 9). These aggressive forms have been shown to correlate with poorer survival outcomes, independent of treatment and disease stage, suggesting that ancestry plays a significant role in tumor biology (10, 11). Recent data illustrate that African American women are disproportionately affected by these aggressive phenotypes, with evidence indicating that approximately 61.9% of endometrial tumors in this group are classified as copy-number-high, compared to only 23.5% in their White counterparts (12, 13). This disparity in molecular profiles aligns with the poor prognosis often assigned to these genetic features, underscoring the urgent need for tailored therapeutic strategies that specifically address these disparities (14, 15).

Furthermore, epigenetic studies have uncovered significant differences in methylation patterns linked to ancestry, revealing that African American tumors have unique methylation markers corresponding to alterations in crucial cancer-related pathways (16). Examination of various differentially methylated CpG sites has implicated diverse regulatory mechanisms that could elucidate the poor prognostic outcomes associated with these aggressive endometrial tumors (17). Against this backdrop, the absence of targeted molecular profiling in African women, particularly in regions such as South Africa, accentuates the complexity involved in understanding the disease and emphasizes the urgent need for research aimed at identifying potential unique drivers operative within this demographic (18, 19).

To address this knowledge gap, the present study aimed to leverage RNA sequencing to establish a foundational transcriptomic dataset derived from formalin-fixed, paraffin-embedded (FFPE) tumors of South African women diagnosed with poorly differentiated endometrial carcinoma. By comparing these findings with existing datasets from African American populations, the research aspires to delineate both shared and unique biological characteristics that could enhance precision oncology approaches, ultimately aiming to improve clinical outcomes for women of African ancestry (7).

In conclusion, the evolving landscape of endometrial carcinoma unveils significant disparities attributed to racial and ethnic backgrounds, driven by the intricate interactions between genetic variations, environmental exposure, and existing health disparities. The promotion of inclusive research efforts that foster the accumulation of population-specific data is critical for developing future therapeutic interventions tailored to encompass diverse demographic contexts (12, 20).

## 2 Materials and methods

### 2.1 Patient cohort and ethics

We retrospectively identified 96 archival FFPE tumors of poorly differentiated endometrial carcinoma from women of African descent treated at Inkosi Albert Luthuli Central Hospital, Durban, South Africa, between 2009 and 2019. The histological subtypes included uterine serous carcinoma and carcinosarcoma, as confirmed by a consultant pathologist.

Demographic and clinicopathological data were obtained from the electronic health records.

Ethics approval was granted by the Biomedical Research Ethics Committee (BREC) of the University of KwaZulu-Natal (protocol reference: BREC/00007085/2024).

### 2.2 Tissue handling and RNA extraction

FFPE tissue blocks were sectioned using an autocut automated microtome (Leica Biosystems, Nussloch, Germany). Tumor-rich regions (>50% tumor content) were selected for RNA extraction and sequencing. Total RNA was extracted using the Quick-RNA™ FFPE Kit (R1008, Zymo Research, Irvine, CA, USA). RNA concentration and purity were assessed using NanoDrop spectrophotometry (A260/280 and A260/230 ratios). RNA integrity was measured using a fragment analyzer (Agilent Technologies, Santa Clara, CA, USA), and sequencing suitability was determined using DV200 values (percentage of RNA fragments ≥200 nucleotides).

### 2.3 Sample inclusion for sequencing and library preparation

Of the 96 archival tumors, 76 samples met the DV200 threshold (≥30%) and were subjected to library preparation and sequencing. For these quality control (QC)-passed samples, sequencing generated sufficient depth for transcriptomic profiling, with each sample yielding millions of paired-end reads and thousands of expressed genes detected, consistent with expectations for FFPE-derived total RNA-seq libraries.

### 2.4 Library preparation and sequencing

RNA libraries were prepared from the 76 QC-passed samples using the NEBNext Single Cell/Low Input RNA Library Prep Kit (New England Biolabs, Ipswich, MA, USA) with modifications for FFPE-derived RNA. Libraries were quantified on a high-sensitivity Bioanalyzer chip, converted to an Onso™-compatible format using the PacBio Onso™ Library Conversion Kit (PacBio, , Menlo Park, CA, USA, 102-529-500), and sequenced on the PacBio Onso™ [Pacific Biosciences (PacBio), Menlo Park, CA, USA] short-read platform with paired-end reads. All samples were sequenced as single biological replicates.

### 2.5 Quality control and read processing

Raw reads were assessed for quality metrics [base quality, gene count (GC) content, and adapter contamination] using FastQC within Galaxy Europe (v25.0). Adapters and low-quality bases were trimmed with Trimmomatic (*SLIDINGWINDOW:4:20* and *MINLEN:36*). Cleaned reads were aligned to the GRCh38 reference genome using minimap2, generating BAM alignment files. Gene-level read assignments were obtained using feature Counts (annotation: hg38.refGene.gtf), and transcript-level abundances were estimated using StringTie to produce TPM matrices for splicing analysis.

### 2.6 Quantification, differential expression, and splicing analysis

Downstream analyses were performed on Galaxy Europe (v25.0) and supplemented with custom Python scripts (Pandas, NumPy, and Matplotlib). Sample 11631/19QT was designated as the internal reference (control) for all transcriptomic comparisons. This sample met all RNA quality and sequencing criteria and exhibited stable global expression patterns. Each tumor sample was compared independently to this control on a gene-by-gene and transcript-by-transcript basis, with expression differences reported as log_2_ fold-change values.

#### 2.6.1 Gene-level quantification

Raw count tables were generated using feature Counts (hg38.refGene.gtf).

#### 2.6.2 Transcript-level quantification

StringTie provided transcript abundance estimates (TPM) and annotations.

#### 2.6.3 Differential expression

Because each tumor sample was sequenced as a single biological replicate (n = 1), variance estimation and statistical hypothesis testing (including p-values and false discovery rate correction) were not applicable and were therefore not performed. Differential expression was instead quantified using log_2_ counts per million (log_2_CPM) normalization and log_2_ fold-change (log_2_FC) relative to the control sample. Genes and transcripts were prioritized based on effect-size thresholds (|log_2_FC| ≥ 0.5–3.0), an approach that minimizes false-positive inference and frames the analysis as exploratory and hypothesis-generating.

#### 2.6.4 Alternative splicing

SUPPA2 was used to generate splicing event definitions [inclusion of exon (IOE) event file] and calculate the percent spliced-in (PSI) values from TPMs. Splicing differences were quantified as ΔPSI between tumor and control samples, prioritizing events in cancer-associated pathways [DNA repair, cell cycle, and epithelial -mesenchymal transition (EMT)].

#### 2.6.5 Visualization and integration

Count, TPM, and PSI matrices were merged using Galaxy utilities and Python. Figures included volcano plots, heatmaps, lollipop plots, bar plots, and Venn diagrams, which were exported as high-resolution vector graphics.

#### 2.6.6 Pathway enrichment

Gene sets derived from high-effect genes and splicing-affected transcripts were evaluated against the Reactome and MSigDB Hallmark pathways. Enrichment was interpreted descriptively because of the absence of replication.

### 2.7 Variability and visualization

Global expression variability was examined across the log_2_FC values. The 30 most variable genes were visualized as a heatmap (pheatmap and R). Volcano plots highlighted the high-effect genes, whereas lollipop plots represented splicing alterations. Venn diagrams illustrated gene set overlaps, and bar plots summarized pathway enrichment results.

### 2.8 Pathway and network analysis

Pathway enrichment was performed using ReactomePA and GSEA (MSigDB Hallmark sets). Pathway enrichment analyses were restricted to protein-coding genes, as pseudogenes, long non-coding RNAs, and readthrough transcripts are not comprehensively represented in curated pathway databases and were therefore excluded from functional enrichment testing. Interaction networks were visualized using Cytoscape to highlight the central regulators among dysregulated genes. Enrichment and network interpretations were descriptive and consistent with the single-replicate design.

### 2.9 Alternative splicing analysis

Transcript isoforms were quantified using SUPPA2 with StringTie-derived TPMs. Splicing events were represented as PSI values, and differences were reported as ΔPSI values. Events in genes associated with key cancer processes were prioritized.

### 2.10 Rationale for single-replicate design

Because each condition was represented by a single replicate, the variance could not be reliably estimated. Therefore, statistical testing [p-values and false discovery rate (FDR) correction] was not performed. Analyses were based on effect sizes (log_2_FC for expression and ΔPSI for splicing) and their consistency across related measures (e.g., transcript- vs. gene-level concordance). This approach, although exploratory, provides a hypothesis-generating framework suitable for FFPE-derived RNA-seq with limited replication.

### 2.11 Statistical analyses

All computational analyses were conducted using R (v4.3.0) and Python. Results are reported in terms of fold-change magnitude and biological relevance rather than statistical significance. No direct statistical integration or joint normalization was performed with external cohorts; comparisons to African American datasets were contextual and literature-based rather than derived from merged expression matrices. Consequently, batch correction was not applied.

## 3 Results

### 3.1 Cohort-level expression profiling (effect-size summary)

**Figure 1:**
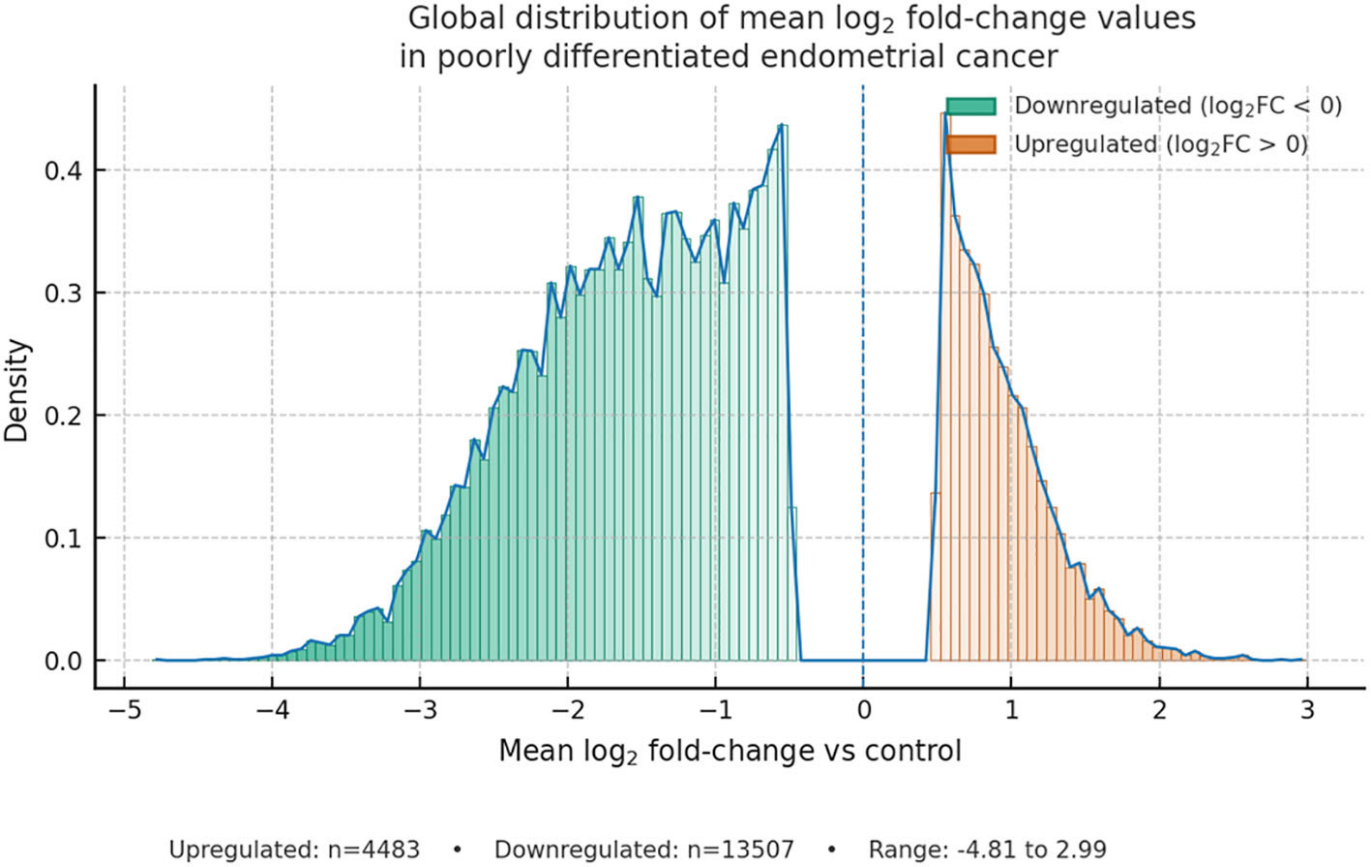
Global distribution of mean log_2_ fold-change values versus control in poorly differentiated endometrial cancer; a cohort-wide histogram with an overlaid density trace depicts transcript-level effects across all tumors, with two-sided gradient bars (white → teal for downregulated and white → orange for upregulated) and a vertical reference at log_2_FC = 0, showing upregulated n = 4,483 and downregulated n = 13,507 within the observed range −4.81 to +2.99.

In the consolidated dataset for poorly differentiated endometrial cancer, 17,990 transcript-level entries exhibited non-zero mean log_2_ fold-change (log_2_FC) values relative to the designated reference sample, comprising 4,483 entries with positive mean log_2_FC and 13,507 entries with negative mean log_2_FC. The observed values spanned from −4.81 to +2.99 across all tumor subgroups (**Figure 1**). These values represent cohort-wide expression differences summarized as effect sizes, rather than statistically tested differential expression (**Figure 1**).

The upregulated component was headed by GJA1 (+2.99; gap-junction/connexin symbol), RNA45SN4 (+2.80; rRNA-annotated locus), DHRSX (+2.62; dehydrogenase/reductase symbol), GRN (+2.60; granulin symbol), ZC3H3 (+2.58; CCCH-type zinc-finger symbol), PCSK4 (+2.55; proprotein convertase symbol), HSPB7 (+2.55; small heat-shock protein symbol), BICRA (+2.49; chromatin-associated symbol), and WASH2P (+2.48; pseudogene “-P” suffix), with mean log_2_FC values as reported (**Table 1**).

**Table 1:** Top 25 upregulated transcripts in poorly differentiated endometrial cancer, listing Gene_ID and mean log_2_ fold-change versus control for the highest positive entries.

*Gene_ID*	Mean_log2FC
*NEB*	9.132207272
*RAPH1*	8.920560719
*MDN1*	8.373434014
*ATXN1*	8.347427589
*RPAP2*	8.344594242
*PHIP*	8.291103083
*PDE4D*	8.170868214
*SOX4*	8.169838166
*GJA1*	2.990200511
*RNA45SN4*	2.803134751
*DHRSX*	2.621048345
*GRN*	2.597142328
*ENSG00000270111*	2.584878349
*ZC3H3*	2.575036273
*PCSK4*	2.55291034
*HSPB7*	2.548888116
*BICRA*	2.488642802
*WASH2P*	2.476049764
*CHEK2P2*	2.475882017
*SPECC1L-ADORA2A*	2.464034235
*P2RY6*	2.423316126
*ZNF837*	2.404099956
*DBF4B*	2.35316518
*FCHO1*	2.314111737
*CCDC120*	2.313773474

The downregulated component showed the most negative mean log_2_FC values for MRPL15 (−4.81; mitochondrial ribosomal protein-like symbol), ZSCAN23 (−4.46; zinc-finger/SCAN-domain symbol), NUF2 (−4.37; kinetochore-associated symbol), RTN1 (−4.33; reticulon-family symbol), EIF3I (−4.31; translation-initiation factor symbol), H1FX-AS1 (−4.28; antisense “-AS1” locus), CSMD2-AS1 (−4.19; antisense “-AS1” locus), HSPB2-C11orf52 (−4.12; readthrough-locus symbol), GLYATL1B (−4.10; glycine-N-acyltransferase-like symbol), and KCNK18 (−4.06; potassium-channel “KCNK” symbol), with values recorded as listed (**Table 2**).

**Table 2:** Top 25 downregulated transcripts in poorly differentiated endometrial cancer, listing Gene_ID and mean log_2_ fold-change versus control for the most negative entries.

*Gene_ID*	Mean_log2FC
*KRTAP9-1*	−9.79079
*TMEM89*	−9.34016
*MAGEH1*	−9.09537
*OR3A2*	−8.10307
*LGALS1*	−8.04807
*OR11H4*	−8.01981
*LOC100288208*	−7.98868
*PRICKLE3*	−7.95196
*NUDT18*	−7.93626
*ATP6V1G3*	−7.91891
*ARMC7*	−7.83947
*LINC00513*	−7.82276
*S100A2*	−7.77171
*LOC101928253*	−7.76693
*FXYD1*	−7.70421
*ENTPD2*	−7.68855
*LOC102724467*	−7.68553
*ZNF628*	−7.6192
*TMEM259*	−7.48985
*PARGP1-AGAP4*	−7.48024
*LINC02546*	−7.3823
*HLA-DPB2*	−7.24535
*SAP30*	−7.16943
*EXOC1L*	−7.11847
*PRAMEF19*	−7.10474

### 3.2 Upregulated transcripts

**Figure 2:**
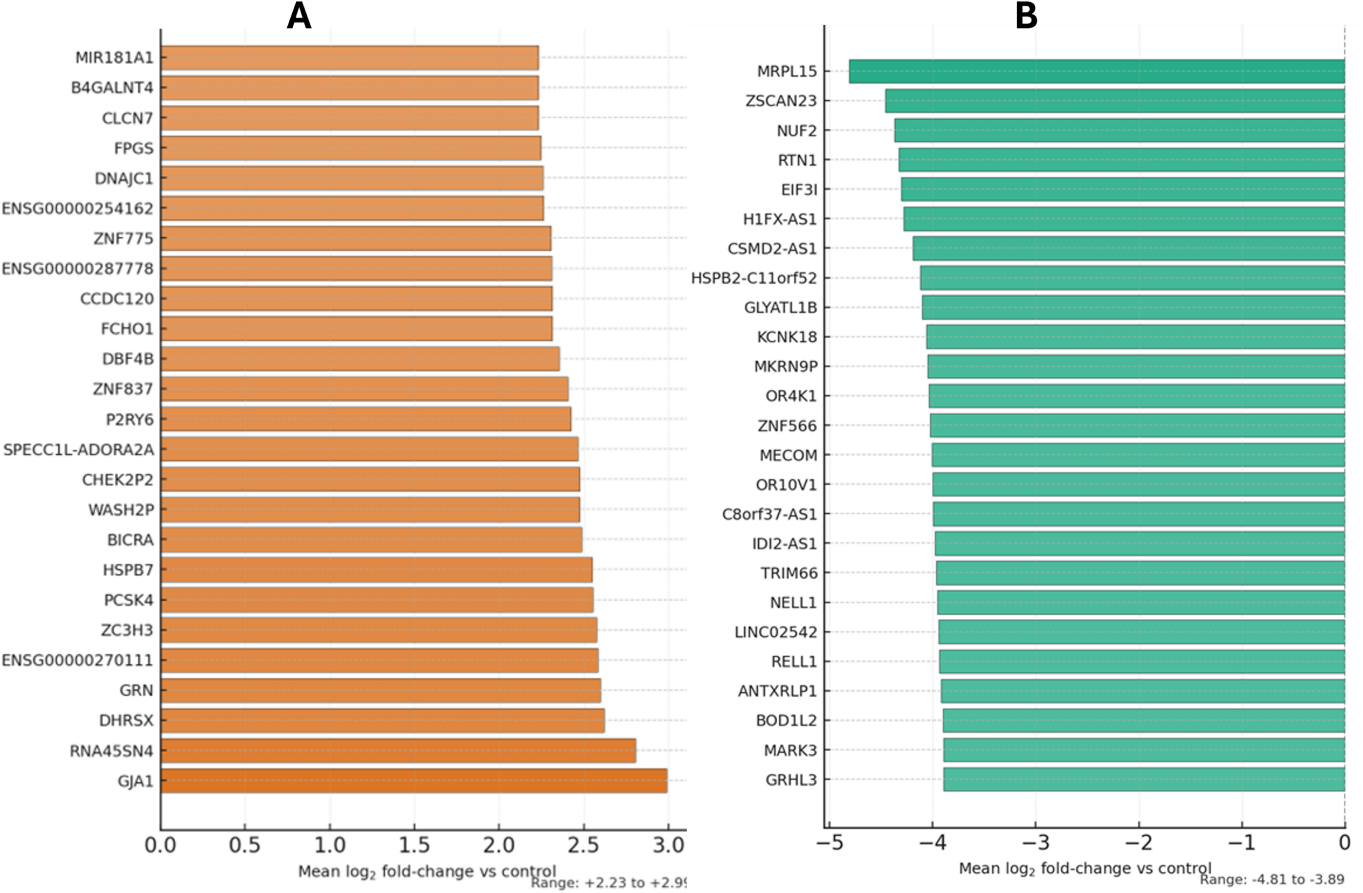
**(A)** Top 25 upregulated transcripts in poorly differentiated endometrial cancer. Horizontal bars represent mean log_2_ fold-change (vs. control), ordered by magnitude. Bar colors follow a continuous white-to-orange gradient scaled by |log_2_FC|, with a vertical reference at 0 marking direction. The strongest increases were observed for *GJA1* (+2.99), *RNA45SN4* (+2.80), and *DHRSX* (+2.62), along with *GRN* (+2.60), *ZC3H3* (+2.58), *PCSK4* (+2.55), *HSPB7* (+2.55), *BICRA* (+2.49), and *WASH2P* (+2.48). **(B)** Top 25 downregulated transcripts in poorly differentiated endometrial cancer. Horizontal bars represent mean log_2_ fold-change (vs. control), ordered by magnitude. Bar colors follow a continuous white-to-teal gradient scaled by |log_2_FC|, with a vertical reference at 0 marking direction. The strongest decreases were observed for *MRPL15* (−4.81), *ZSCAN23* (−4.46), and *NUF2* (−4.37), along with *RTN1* (−4.33), *EIF3I* (−4.31), *H1FX-AS1* (−4.28), *CSMD2-AS1* (−4.19), *HSPB2-C11orf52* (−4.12), *GLYATL1B* (−4.10), and *KCNK18* (−4.06).

Transcript-level expression changes are reported explicitly throughout this section using Ensembl transcript identifiers, readthrough annotations, and long non-coding RNA (lncRNA) locus names; transcript-resolved alterations are further detailed in the Alternative Splicing and Novel Transcript Discovery sections. Within the upregulated direction, the top 25 transcripts showed mean log_2_FC values from +2.99 to +2.23 and comprised protein-coding symbols together with RNA-annotated, pseudogene, readthrough, and Ensembl-labeled loci: GJA1 (+2.99; gap-junction/connexin symbol), RNA45SN4 (+2.80; rRNA-annotated locus), DHRSX (+2.62; dehydrogenase/reductase symbol), GRN (+2.60; granulin symbol), ENSG00000270111 (+2.58; Ensembl-annotated locus), ZC3H3 (+2.58; CCCH-type zinc-finger symbol), PCSK4 (+2.55; proprotein-convertase symbol), HSPB7 (+2.55; small heat-shock protein symbol), BICRA (+2.49; chromatin-associated symbol), WASH2P (+2.48; pseudogene “-P” suffix), CHEK2P2 (+2.48; pseudogene “-P” suffix), SPECC1L-ADORA2A (+2.46; readthrough locus), P2RY6 (+2.42; purinergic-receptor symbol), ZNF837 (+2.40; zinc-finger “ZNF” symbol), DBF4B (+2.35; DBF4-related symbol), FCHO1 (+2.31; FCH-domain symbol), CCDC120 (+2.31; coiled-coil-domain symbol), ENSG00000287778 (+2.31; Ensembl-annotated locus), ZNF775 (+2.31; zinc-finger “ZNF” symbol), ENSG00000254162 (+2.26; Ensembl-annotated locus), DNAJC1 (+2.26; DnaJ/Hsp40-family symbol), FPGS (+2.25; synthase-annotated symbol), CLCN7 (+2.23; chloride-channel symbol), B4GALNT4 (+2.23; glycosyltransferase symbol), and MIR181A1 (+2.23; microRNA locus), with values reported as listed (**Table 1**, **Figure 2A**).

### 3.3 Downregulated transcripts

Within the downregulated direction, the most negative mean log_2_FC values were recorded for MRPL15 (−4.81; mitochondrial ribosomal protein-like symbol), ZSCAN23 (−4.46; zinc-finger/SCAN-domain symbol), NUF2 (−4.37; kinetochore-associated symbol), RTN1 (−4.33; reticulon-family symbol), EIF3I (−4.31; translation-initiation factor symbol), H1FX-AS1 (−4.28; antisense locus), CSMD2-AS1 (−4.19; antisense locus), HSPB2-C11orf52 (−4.12; readthrough locus), GLYATL1B (−4.10; glycine-N-acyltransferase-like symbol), KCNK18 (−4.06; potassium-channel “KCNK” symbol), MKRN9P (−4.05; pseudogene “-P” symbol), OR4K1 (−4.04; olfactory-receptor symbol), ZNF566 (−4.03; zinc-finger “ZNF” symbol), MECOM (−4.01; transcription-related symbol), OR10V1 (−4.00; olfactory-receptor symbol), C8orf37-AS1 (−4.00; antisense locus), IDI2-AS1 (−3.98; antisense locus), TRIM66 (−3.97; tripartite-motif symbol), NELL1 (−3.95; NELL-family symbol), LINC02542 (−3.94; long intergenic non-coding RNA locus), RELL1 (−3.94; receptor-like symbol), ANTXRLP1 (−3.92; “like-protein” symbol), BOD1L2 (−3.90; microtubule-attachment-like symbol), MARK3 (−3.89; microtubule-affinity-regulating kinase symbol), and GRHL3 (−3.89; transcription-related symbol), with values reported exactly as in the results (**Table 2**, **Figure 2B**).

### 3.4 Most downregulated genes and subgroup distribution

From the master expression summary table, the 25 most downregulated transcripts by mean log_2_FC across the 76-sample cohort were as follows: MRPL15 (−4.81; mitochondrial ribosomal protein), ZSCAN23 (−4.46; zinc-finger/SCAN transcription factor), NUF2 (−4.37; kinetochore complex component), RTN1 (−4.33; endoplasmic-reticulum membrane protein), EIF3I (−4.31; translation-initiation factor subunit), H1FX-AS1 (−4.28; antisense long non-coding RNA), CSMD2-AS1 (−4.19; antisense long non-coding RNA), HSPB2-C11orf52 (−4.12; readthrough transcript), GLYATL1B (−4.10; acyltransferase-like enzyme), KCNK18 (−4.06; two-pore potassium channel), MKRN9P (−4.05; pseudogene), OR4K1 (−4.04; olfactory receptor), ZNF566 (−4.03; zinc-finger transcription factor), MECOM (−4.01; transcription factor), OR10V1 (−4.00; olfactory receptor), C8orf37-AS1 (−3.999; antisense long non-coding RNA), IDI2-AS1 (−3.98; antisense long non-coding RNA), TRIM66 (−3.97; tripartite-motif protein), NELL1 (−3.95; EGF-like secreted protein), LINC02542 (−3.94; long intergenic non-coding RNA), RELL1 (−3.94; TNF-receptor-family-like protein), ANTXRLP1 (−3.92; pseudogene), BOD1L2 (−3.90; BOD1-like paralog), MARK3 (−3.89; serine/threonine kinase), and GRHL3 (−3.89; transcription factor) (**Table 2**).

**Figure 3:**
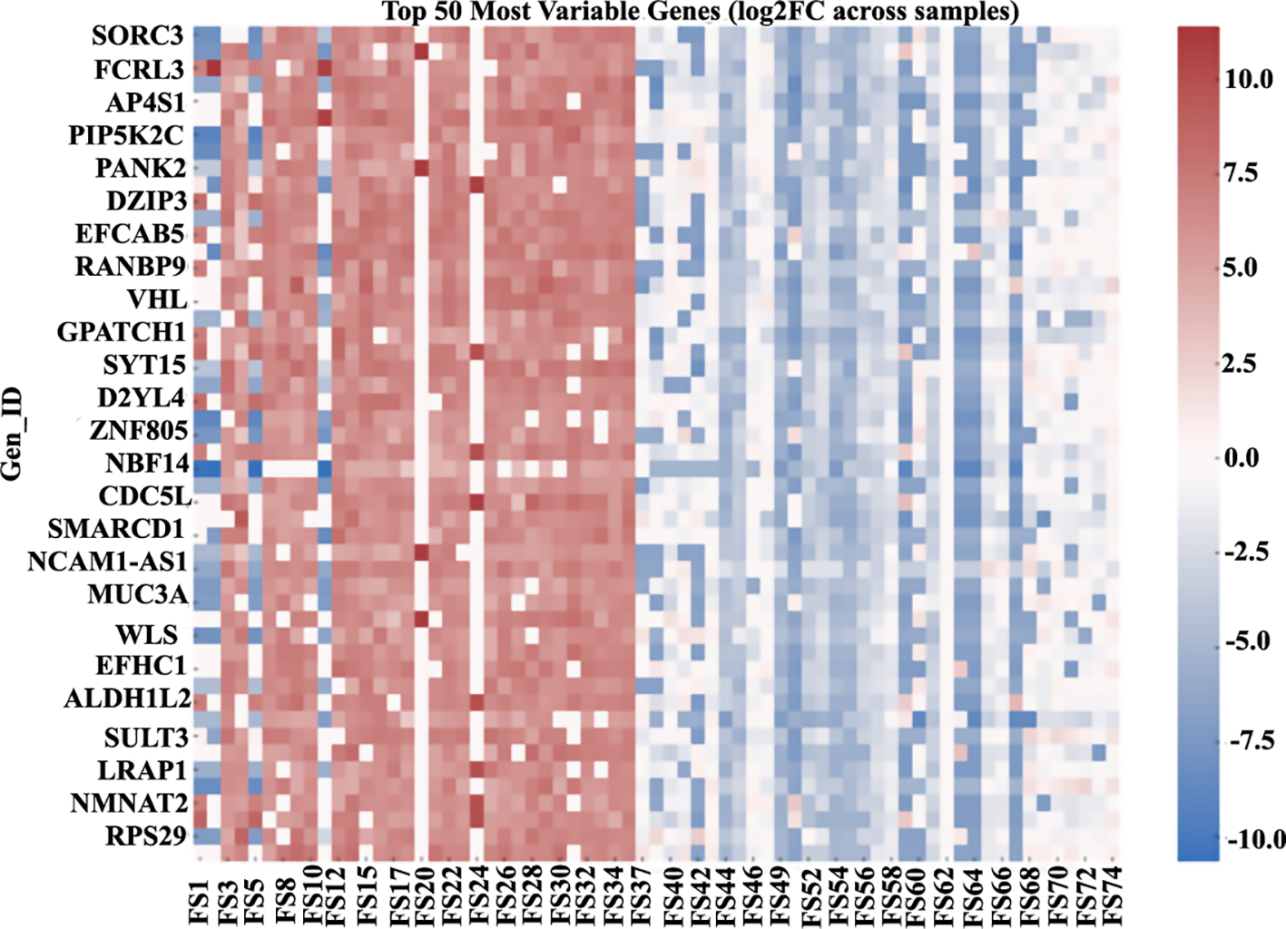
Heatmap of the top 50 most variable transcripts across 76 tumors; consensus clustering of log_2_FC values depicts sample-wise and transcript-wise structures with prominent upregulated clusters (e.g., *MKNK2*, *RARA*, and *SIX5*) and broadly downregulated zinc-finger genes (e.g., *ZNF793*, *ZNF569*, and *ZNF382*). The heatmap illustrates relative expression variability based on log_2_ fold-change values and is not intended to convey statistical significance. Samples are shown as columns and grouped by unsupervised clustering; group-level structure is inferred from clustering rather than predefined labels.

Beyond the top 25 list, several cancer-relevant regulators in the master table also showed pronounced negative fold-changes, including CDC73 (−3.36; transcriptional regulator, PAF1 complex), MYC (−2.96; transcription factor), and FRS2 (−2.90; FGFR adaptor), together with a group of zinc-finger transcription factors: ZNF569 (−3.36), ZNF573 (−2.92), ZNF528 (−2.74), ZNF606 (−2.26), ZNF382 (−1.87), and ZNF793 (−2.33). The table also records NBPF14 (−1.23; NBPF-family protein), LINC01193 (−1.48; long non-coding RNA), and LINC01605 (−0.62; long non-coding RNA). For comparison, the strongest positive fold-changes in the table comprised DHRSX (+2.62; oxidoreductase), BICRA (+2.49; chromatin regulator), LINC02692 (+2.23; long non-coding RNA), LMF1-AS1 (+1.91; antisense long non-coding RNA), MGAT3 (+1.54; glycosyltransferase), PRR5 (+1.47; mTORC2-associated regulator), SIX5 (+1.43; homeobox transcription factor), and RARA (+0.76; nuclear receptor) (**Figure 2B**). The variability and clustering of the top 50 most variable transcripts across all samples are illustrated in **Figure 3**.

**Figure 4:**
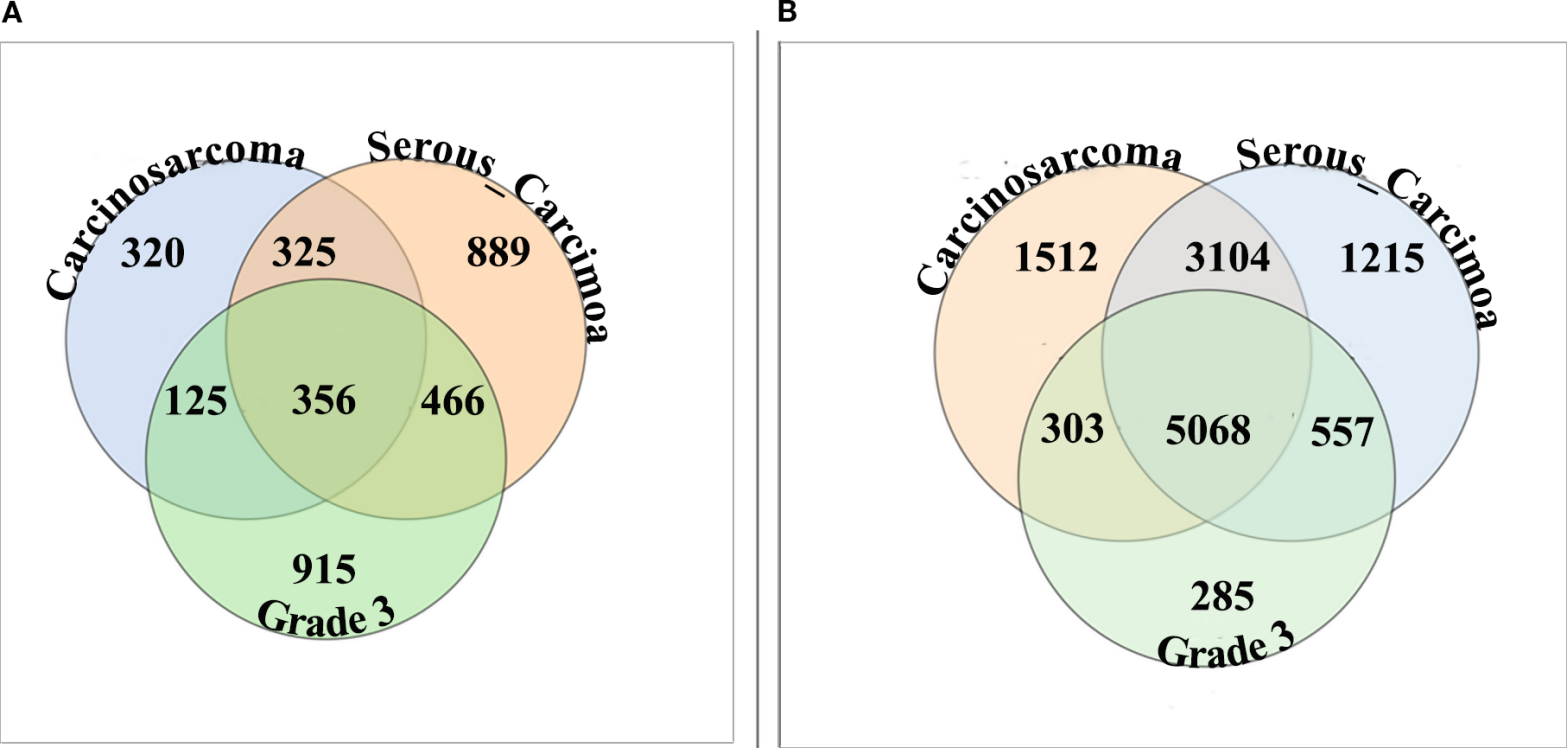
Overlap of showing non-zero mean log_2_FC genes across subgroups. **(A)** Upregulated Venn with the central intersection of 356. **(B)** Downregulated Venn with the central intersection of 5,068.

When stratified by histology, the bar plot summary built at the |log_2_FC| ≥ 1.0 threshold showed that downregulated genes outnumbered upregulated genes in carcinosarcoma, serous carcinoma, and grade 3 tumors (**Figure 4**). Inter-subgroup overlap analysis at the same threshold identified 356 transcripts commonly upregulated across all three subgroups, with additional subgroup-restricted upregulated sets of 320 (carcinosarcoma only), 889 (serous carcinoma only), and 915 (grade 3 only), and pairwise upregulated overlaps of 325 (carcinosarcoma/serous), 125 (carcinosarcoma/grade 3), and 466 (serous/grade 3). Conversely, 5,068 transcripts were commonly downregulated across all subgroups, alongside subgroup-restricted downregulated sets of 1,512 (carcinosarcoma only), 1,215 (serous carcinoma only), and 285 (grade 3 only), with pairwise downregulated overlaps of 3,104 (carcinosarcoma/serous), 303 (carcinosarcoma/grade 3), and 557 (serous/grade 3).

### 3.5 Pathway enrichment analysis

**Figure 5:**
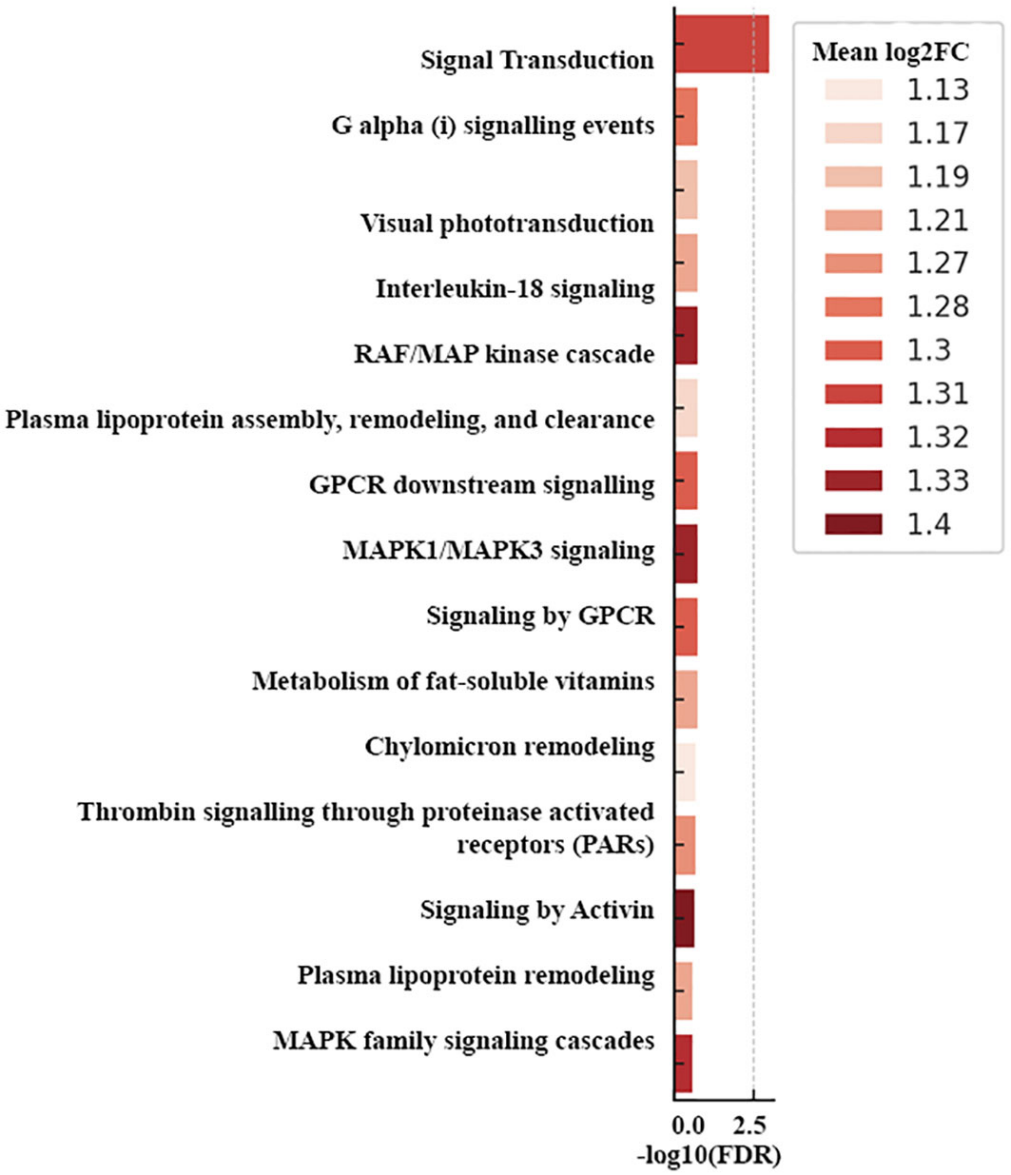
Up-reactome. Top reactome pathways enriched in the upregulated gene set from 76 cases of p-differentiated endometrial cancers (|log_2_FC| ≥ 1.0), shown as horizontal bars ranked by –log_10_(FDR) with bar color encoding the pathway-level mean log_2_FC (+1.13 to +1.40). FDR, false discovery rate.

For functional interpretation, enrichment analyses were restricted to protein-coding genes derived from the expression summary table, while non-coding transcripts were reported separately in transcript-level, splicing, and novel isoform analyses. Reactome over-representation using transcripts filtered at |log_2_FC| ≥ 1.0 identified coherent pathway-level signals in the 76-sample cohort. The upregulated enrichment profile was led by signal transduction categories, including signal transduction, G alpha (i) signaling events, GPCR downstream signaling, signaling by GPCR, RAF/MAP kinase cascade, MAPK1/MAPK3 signaling, MAPK family signaling cascades, interleukin-18 signaling, thrombin signaling through proteinase-activated receptors (PARs), signaling by activin, and lipid-handling pathways (plasma lipoprotein assembly, remodeling, and clearance, plasma lipoprotein remodeling, chylomicron remodeling, and metabolism of fat-soluble vitamins), with mean log_2_FC values in the enriched bars spanning approximately +1.13 to +1.40. Representative upregulated genes from the master table mapping to these modules included MKNK2 (+2.03; MAPK-interacting serine/threonine kinase driving translational control), BICRA (+2.49; chromatin regulator), ZNF837 (+2.40; zinc-finger transcription factor), SIX5 (+1.43; homeobox transcription factor), RARA (+0.76; nuclear receptor), FGF2 (+1.70; growth factor), DOCK1 (+1.69; Rac guanine-nucleotide exchange factor involved in cytoskeletal remodeling), and MGAT3 (+1.54; N-glycan β-1,4-GlcNAc transferase), consistent with the gene-level increases recorded in the expression summary table (**Figure 5**).

**Figure 6:**
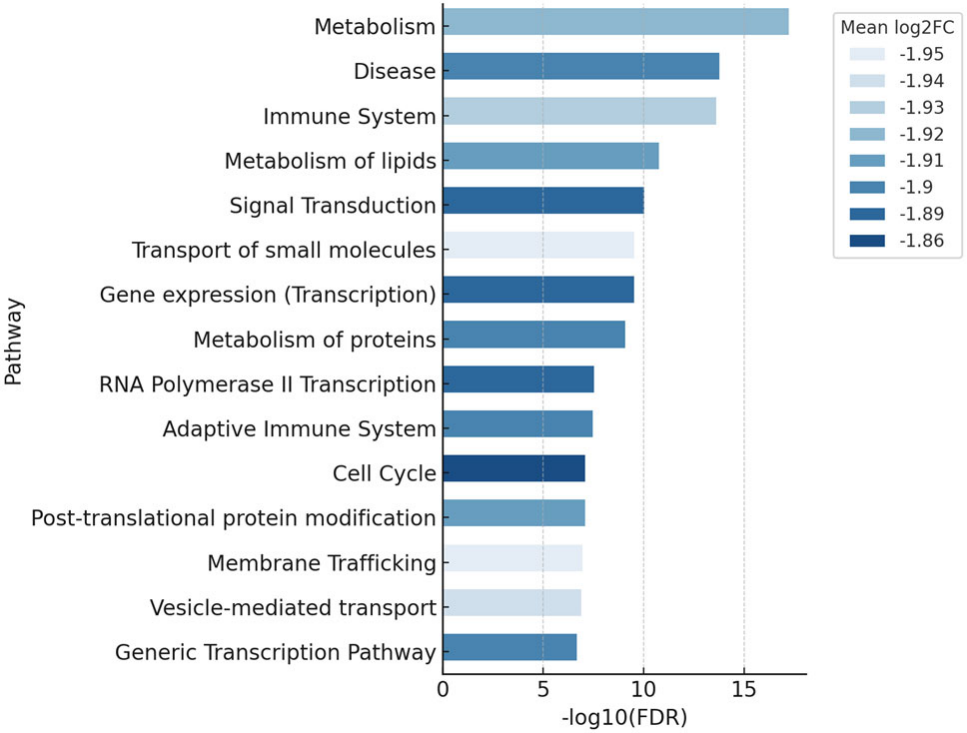
Down-reactome. Top reactome pathways enriched in the downregulated gene set from the same cohort (|log_2_FC| ≤ −1.0), shown as horizontal bars ranked by −log_10_(FDR), with bar color encoding the pathway-level mean log_2_FC (−1.95 to −1.86). FDR, false discovery rate.

The downregulated enrichment profile showed broad suppression across foundational cellular programs, with the strongest bars spanning approximately −1.95 to −1.86 in mean log_2_FC and including metabolism, disease, immune system, metabolism of lipids, signal transduction, transport of small molecules, gene expression (transcription), metabolism of proteins, RNA polymerase II transcription, adaptive immune system, cell cycle, post-translational protein modification, membrane trafficking, vesicle-mediated transport, and generic transcription pathway. Leading downregulated genes from the table linked to these categories included MYC (−2.96; transcription factor), NUF2 (−4.37; kinetochore complex component), KIF20A (−3.66; mitotic kinesin), MCM3 (−3.47; DNA replication licensing factor), CENPA (−3.50; centromeric histone variant), EGFR (−1.38; receptor tyrosine kinase), ERBB3 (−1.52; receptor tyrosine kinase), PIK3R1 (−2.44; class IA PI3K regulatory subunit), PIK3CA (−1.34; class IA PI3K catalytic subunit), ITGA3 (−3.11; integrin α subunit), ITGB1 (−2.51; integrin β subunit), ITGB5 (−2.21; integrin β subunit), THBS1 (−2.83; adhesive glycoprotein), COL4A3 (−2.54; basement-membrane collagen), VWF (−2.77; multimeric adhesion glycoprotein), FBN1 (−3.01; extracellular-matrix glycoprotein), JAM2 (−2.81; junctional adhesion molecule), JAM3 (−2.59; junctional adhesion molecule), MMP14 (−3.15; membrane-type metalloproteinase), TIMP2 (−3.18; metalloproteinase inhibitor), and EPHA2 (−1.90; Eph receptor tyrosine kinase) (depicted in **Figure 6**, **Tables 3**, **4**).

**Table 3:** Upregulated reactome.

Pathway	Regulation	neglog10FDR	FDR	Mean_log2FC	Overlap	Leading genes (top 8 by |log2FC|)
Signal transduction	Upregulated	2.974694	1.06E−03	1.31	200	GJA1 (+2.99); IL3RA (+2.13); APBB1IP (+1.98); DOCK6 (+1.95); GNB4 (+1.91); ARHGEF18 (+1.90); SAMM50 (+1.86); CUL1 (+1.86)
G alpha (i) signaling events	Upregulated	0.739929	1.82E−01	1.28	33	GNB4 (+1.91); S1PR4 (+1.77); PPP1R1B (+1.74); CAMK2B (+1.60); GALR1 (+1.58); GPSM1 (+1.51); CXCR3 (+1.49); BDKRB2 (+1.47)
Visual phototransduction	Upregulated	0.739929	1.82E−01	1.19	15	SDC3 (+1.60); OPN1LW (+1.43); APOE (+1.33); GPC6 (+1.28); GNB5 (+1.21); FNTA (+1.18); OPN1MW (+1.16); AGRN (+1.14)
Interleukin-18 signaling	Upregulated	0.739929	1.82E−01	1.21	4	ALOX5 (+1.42); IL18R1 (+1.26); IL13 (+1.11); IL18RAP (+1.05)
RAF/MAP kinase cascade	Upregulated	0.739929	1.82E−01	1.33	28	IL3RA (+2.13); APBB1IP (+1.98); GRIN2D (+1.82); GFRA2 (+1.81); FGF2 (+1.70); KBTBD7 (+1.61); CAMK2B (+1.60); HBEGF (+1.58)
Plasma lipoprotein assembly, remodeling, and clearance	Upregulated	0.739929	1.82E−01	1.17	12	CREB3L3 (+1.75); APOE (+1.33); PRKACG (+1.18); LMF2 (+1.13); AP2A2 (+1.13); AMN (+1.09); GPIHBP1 (+1.07); NCEH1 (+1.07)
GPCR downstream signaling	Upregulated	0.739929	1.82E−01	1.3	54	GNB4 (+1.91); ARHGEF18 (+1.90); ADRB3 (+1.81); GRK6 (+1.78); S1PR4 (+1.77); PPP1R1B (+1.74); ARHGEF4 (+1.62); CAMK2B (+1.60)
MAPK1/MAPK3 signaling	Upregulated	0.739929	1.82E−01	1.33	28	IL3RA (+2.13); APBB1IP (+1.98); GRIN2D (+1.82); GFRA2 (+1.81); FGF2 (+1.70); KBTBD7 (+1.61); CAMK2B (+1.60); HBEGF (+1.58)
Signaling by GPCR	Upregulated	0.739929	1.82E−01	1.3	59	GNB4 (+1.91); ARHGEF18 (+1.90); ADRB3 (+1.81); GRK6 (+1.78); S1PR4 (+1.77); PPP1R1B (+1.74); FZD1 (+1.74); ARHGEF4 (+1.62)
Metabolism of fat-soluble vitamins	Upregulated	0.739929	1.82E−01	1.21	9	SDC3 (+1.60); APOE (+1.33); VKORC1 (+1.33); GPC6 (+1.28); AGRN (+1.14); GPIHBP1 (+1.07); LPL (+1.06); APOC3 (+1.05)

Reactome pathway enrichment results for the upregulated gene set (|log_2_FC| ≥ 1.0; 76 tumors), ranked by −log_10_(FDR), reporting columns: Pathway, −log_10_(FDR), FDR, Mean log_2_FC, Overlap, and Leading genes (top 8 by |log_2_FC| with their log_2_FC values).

FDR, false discovery rate.

**Table 4:** Downregulated reactome.

Pathway	Regulation	neglog10FDR	FDR	Mean_log2FC	Overlap	Leading genes (top 8 by |log2FC|)
Metabolism	Downregulated	17.22768	5.92E−18	−1.92	963	UQCRFS1 (−3.82); COMT (−3.74); NUP37 (−3.74); HSD17B10 (−3.72); ATP5PO (−3.58); CYP4A11 (−3.58); ADH4 (−3.57); ACSM6 (−3.56)
Disease	Downregulated	13.78781	1.63E−14	−1.9	813	MARK3 (−3.89); STAM (−3.77); COMT (−3.74); NUP37 (−3.74); IFNA8 (−3.63); CTBP2 (−3.53); STX1A (−3.52); GNG2 (−3.48)
Immune system	Downregulated	13.63078	2.34E−14	−1.93	889	BPIFA2 (−3.85); IL15 (−3.84); NUP37 (−3.74); HTN3 (−3.71); KIF20A (−3.66); IFNA8 (−3.63); TMEM63A (−3.59); AGA (−3.58)
Metabolism of lipids	Downregulated	10.75696	1.75E−11	−1.91	365	CYP4A11 (−3.58); ACSM6 (−3.56); HMGCLL1 (−3.52); ARNTL (−3.39); ARF1 (−3.35); ACSBG2 (−3.27); HPGDS (−3.20); ELOVL4 (−3.18)
Signal transduction	Downregulated	10.04721	8.97E−11	−1.89	1084	NUF2 (−4.37); MECOM (−4.01); MARK3 (−3.89); STAM (−3.77); NUP37 (−3.74); NLK (−3.70); ADH4 (−3.57); ID2 (−3.57)
Transport of small molecules	Downregulated	9.533132	2.93E−10	−1.95	346	SLC22A3 (−3.54); GNG2 (−3.48); ARF1 (−3.35); ABCG1 (−3.32); ALAD (−3.19); SLC22A4 (−3.18); UBA52 (−3.17); CTNS (−3.15)
Gene expression (transcription)	Downregulated	9.519993	3.02E−10	−1.89	679	ZNF566 (−4.03); NUP37 (−3.74); CNOT3 (−3.44); KRBOX4 (−3.44); CHTOP (−3.44); ZNF570 (−3.43); ARNTL (−3.39); ZNF268 (−3.37)
Metabolism of proteins	Downregulated	9.079877	8.32E−10	−1.9	865	MRPL15 (−4.81); EIF3I (−4.31); STAM (−3.77); NUP37 (−3.74); HSD17B10 (−3.72); ST8SIA1 (−3.68); APOL1 (−3.63); NAPB (−3.61)
RNA polymerase II transcription	Downregulated	7.540608	2.88E−08	−1.89	581	ZNF566 (−4.03); CNOT3 (−3.44); KRBOX4 (−3.44); CHTOP (−3.44); ZNF570 (−3.43); ARNTL (−3.39); ZNF268 (−3.37); ZNF569 (−3.36)
Adaptive immune system	Downregulated	7.476254	3.34E−08	−1.9	353	KIF20A (−3.66); HLA−DOB (−3.57); LILRB1 (−3.48); RNF111 (−3.35); ARF1 (−3.35); TAB2 (−3.23); UBA52 (−3.17); RNF115 (−3.15)

Reactome pathway enrichment results for the downregulated gene set (|log_2_FC| ≤ −1.0; 76 tumors), ranked by −log_10_(FDR), reporting columns: Pathway, −log_10_(FDR), FDR, Mean log_2_FC, Overlap, and Leading genes (top 8 by |log_2_FC| with their log_2_FC values).

FDR, false discovery rate.

### 3.6 Alternative splicing analysis

**Figure 7:**
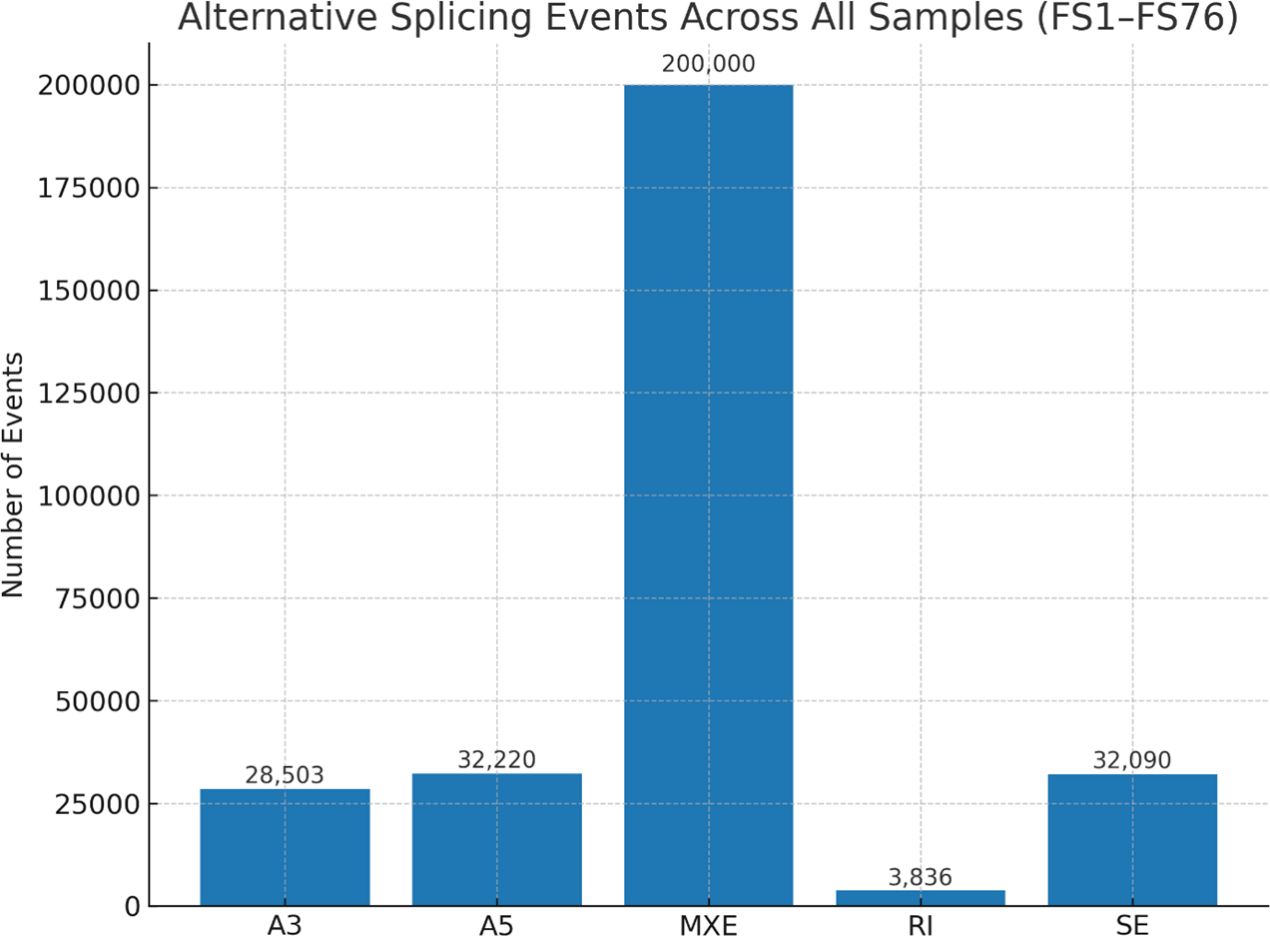
Bar chart of total alternative splicing events (MXE, A5, SE, A3, and RI) across 76 tumors; counts are aggregated across samples and indicated in the panel. MXE, mutually exclusive exon; A5, alternative 5′ splice site; SE, skipped exon; A3, alternative 3′ splice site; RI, retained intron.

Across the unified cohort (n = 76), the total alternative splicing event counts consisted of mutually exclusive exons (MXE; ~200,000), alternative 5′ splice sites (A5; 32,220), skipped exons (SE; 32,090), alternative 3′ splice sites (A3; 28,503), and retained introns (RI; 3,836), summarizing the event landscape used for downstream comparisons (**Figure 7**).

**Figure 8:**
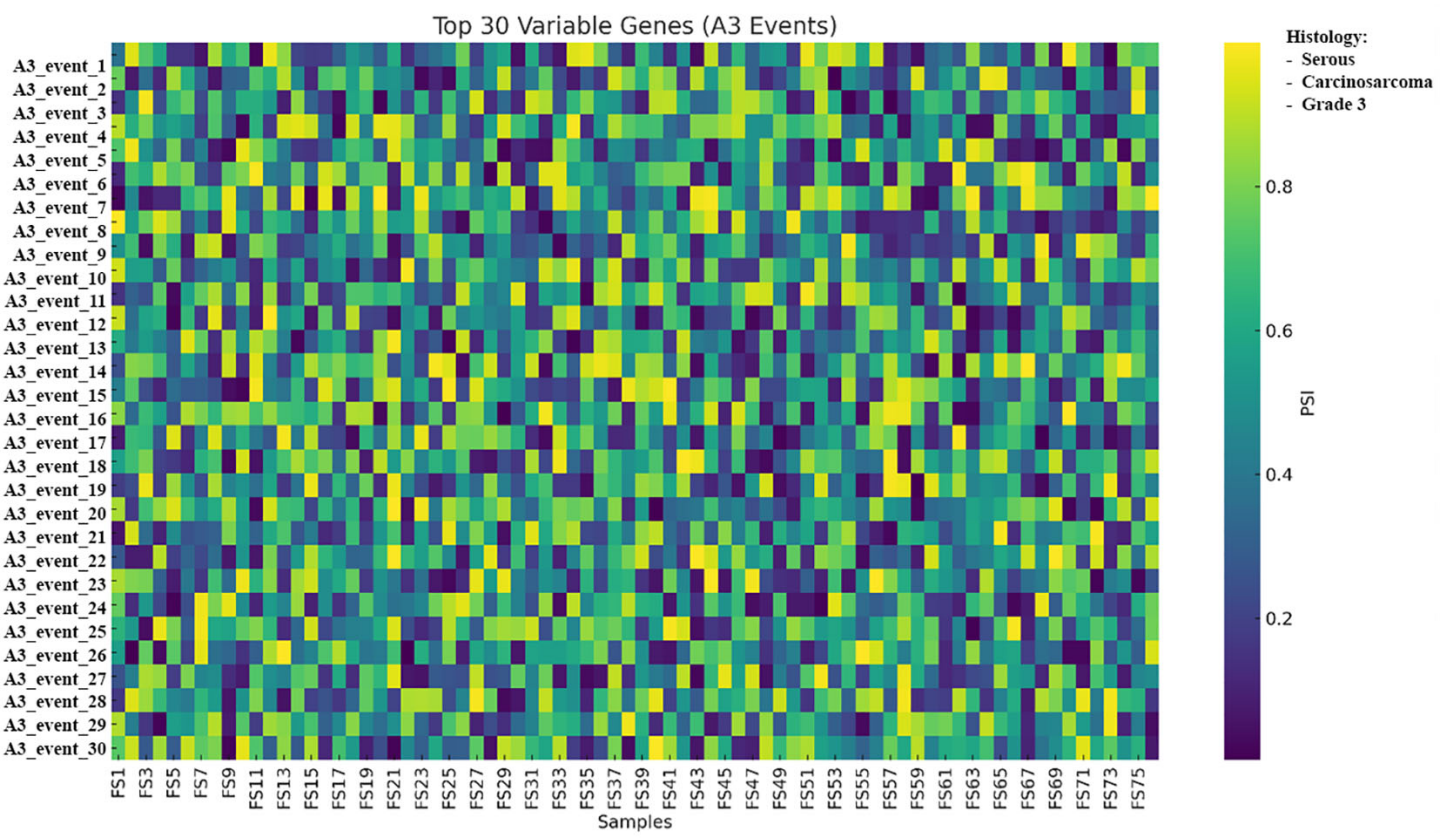
Heatmap of the 30 most variable A3 alternative splicing events across 76 tumors; rows represent SUPPA2-defined splicing events, columns represent samples, and color denotes PSI from low to high. PSI values represent exon inclusion levels per sample and are shown to illustrate relative splicing variability across tumors; no statistical testing was applied due to the single-replicate design. Samples are shown as columns and grouped by unsupervised clustering of PSI values; histological subtype (serous, carcinosarcoma, and grade 3) is indicated by the annotation bar. A3, alternative 3′ splice site; PSI, percent spliced-in.

A cohort-level heatmap of A3 events showed the 30 highest-variance genes across the 76 samples, with rows representing genes and columns representing tumors, colored according to PSI (**Figure 8**).

**Figure 9:**
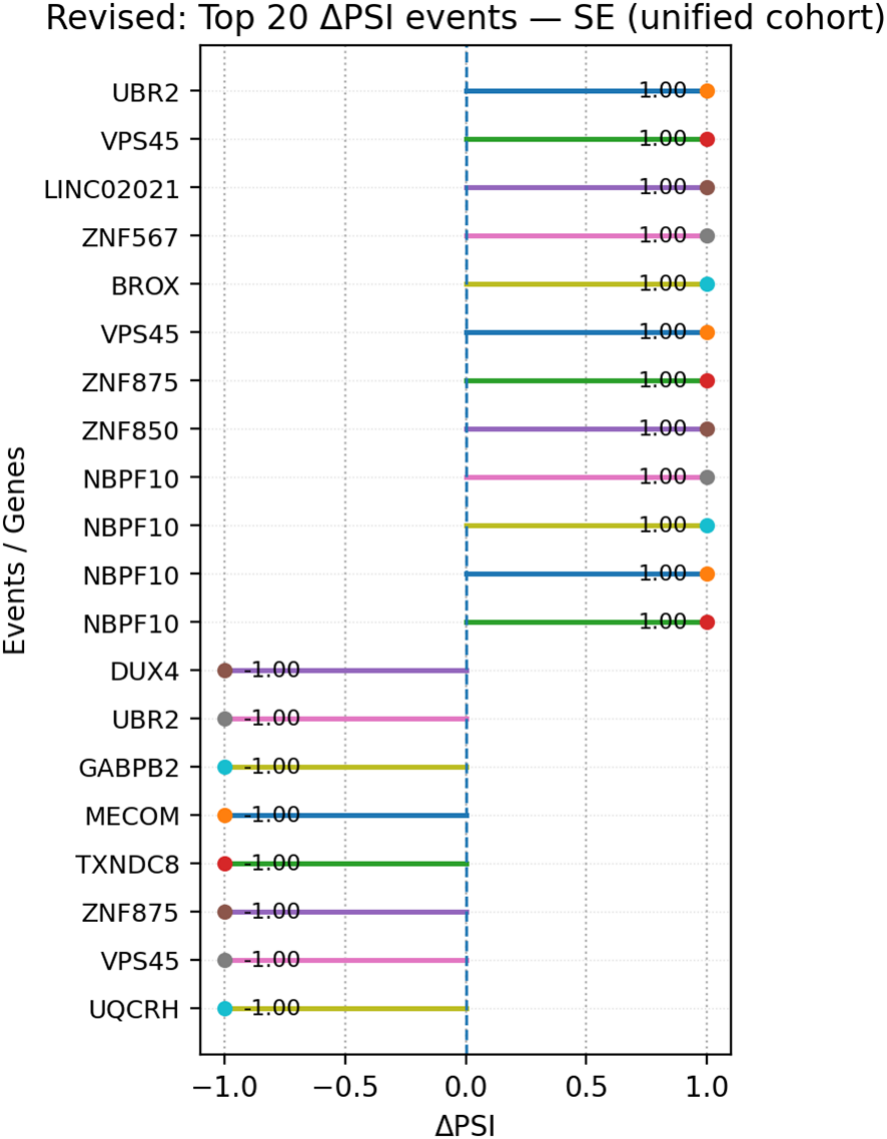
Lollipop plot of the top 20 SE events ranked by |ΔPSI| in the unified cohort; segments indicate ΔPSI magnitude per event. Events are ranked by absolute ΔPSI as an effect-size measure; multiple top-ranked events may display identical or boundary ΔPSI values (e.g., ± 1.0), reflecting near-complete exon inclusion or exclusion rather than uniform splicing behavior. SE, skipped exon; PSI, percent spliced-in.

Within SE events, genes present in the top 20 by |ΔPSI| and recorded in the expression summary table included UBR2 (log_2_FC −1.88; E3 ubiquitin ligase), NBPF10 (−1.90; NBPF-family protein), DUX4 (−0.79; double-homeobox transcription factor), GABPB2 (−2.00; ETS-family transcription factor subunit), MECOM (−4.01; transcription factor), and TXNDC8 (−1.34; thioredoxin-domain protein) (**Figure 9**).

**Figure 10:**
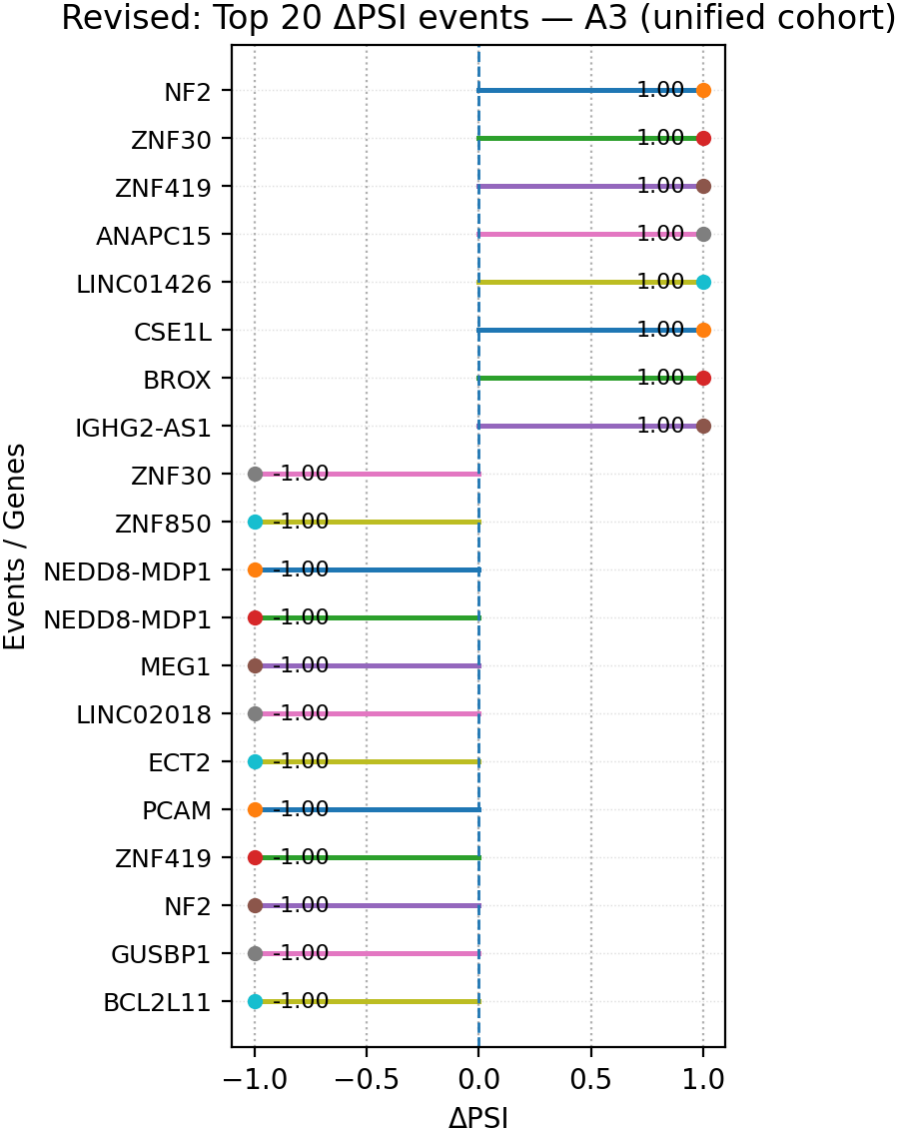
Lollipop plot of the top 20 A3 events ranked by |ΔPSI| in the unified cohort; segments indicate the ΔPSI magnitude per event. Events are ranked by absolute ΔPSI as an effect-size measure; multiple top-ranked events may display identical or boundary ΔPSI values (e.g., ± 1.0), reflecting near-complete exon inclusion or exclusion rather than uniform splicing behavior. A3, alternative 3′ splice site; PSI, percent spliced-in.

Within A3 events, genes present in the top 20 by |ΔPSI| and in the expression summary table included NF2 (−0.79; tumor suppressor), ZNF30 (−0.61; zinc-finger transcription factor), ZNF419 (−1.32; zinc-finger transcription factor), ANAPC15 (+0.56; anaphase-promoting complex subunit), CSE1L (−2.33; nuclear export factor), BROX (−2.10; ESCRT-associated protein), HMGA2-AS1 (−2.50; antisense long non-coding RNA), ZNF850 (−1.42; zinc-finger transcription factor), NEDD8-MDP1 (+0.61; readthrough locus), MEIG1 (−1.56; meiosis-associated protein), ECT2 (−1.89; Rho guanine-nucleotide exchange factor), PGAM5 (−0.90; mitochondrial phosphatase), GUSBP1 (−1.77; pseudogene locus), and BCL2L11 (−1.87; apoptosis regulator) (**Figure 10**).

**Figure 11:**
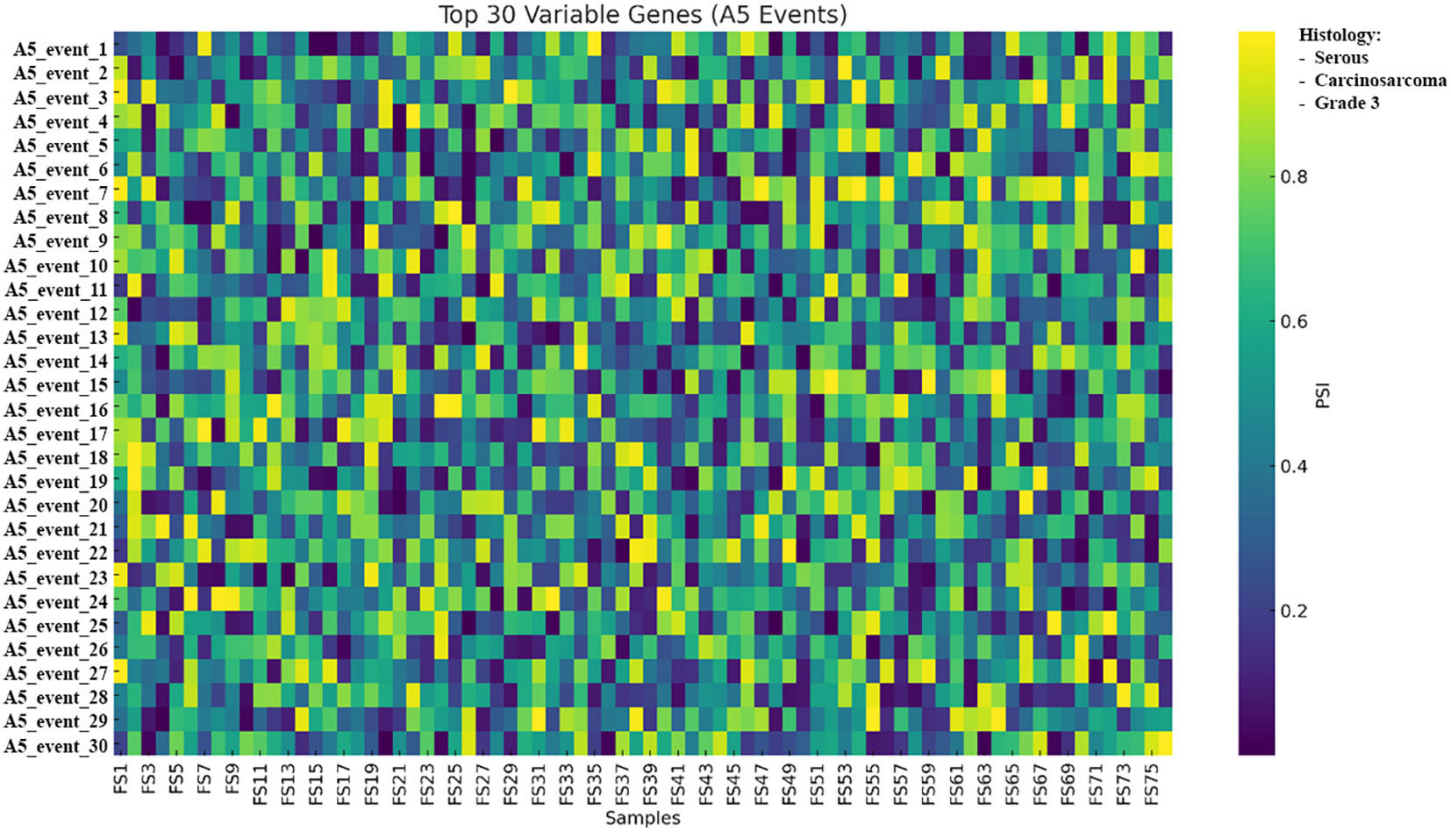
Heatmap of the 30 most variable A5 alternative splicing events across 76 tumors; rows represent SUPPA2-defined splicing events, columns represent samples, and color denotes PSI from low to high. PSI values represent exon inclusion levels per sample and are shown to illustrate relative splicing variability across tumors; no statistical testing was applied due to the single-replicate design. Samples are shown as columns and grouped by unsupervised clustering of PSI values; histological subtype (serous, carcinosarcoma, and grade 3) is indicated by the annotation bar. A5, alternative 5′ splice site; PSI, percent spliced-in.

A heatmap of A5 events displayed the 30 genes with the highest variance across the cohort, using PSI-based coloring, as described above (**Figure 11**).

**Figure 12:**
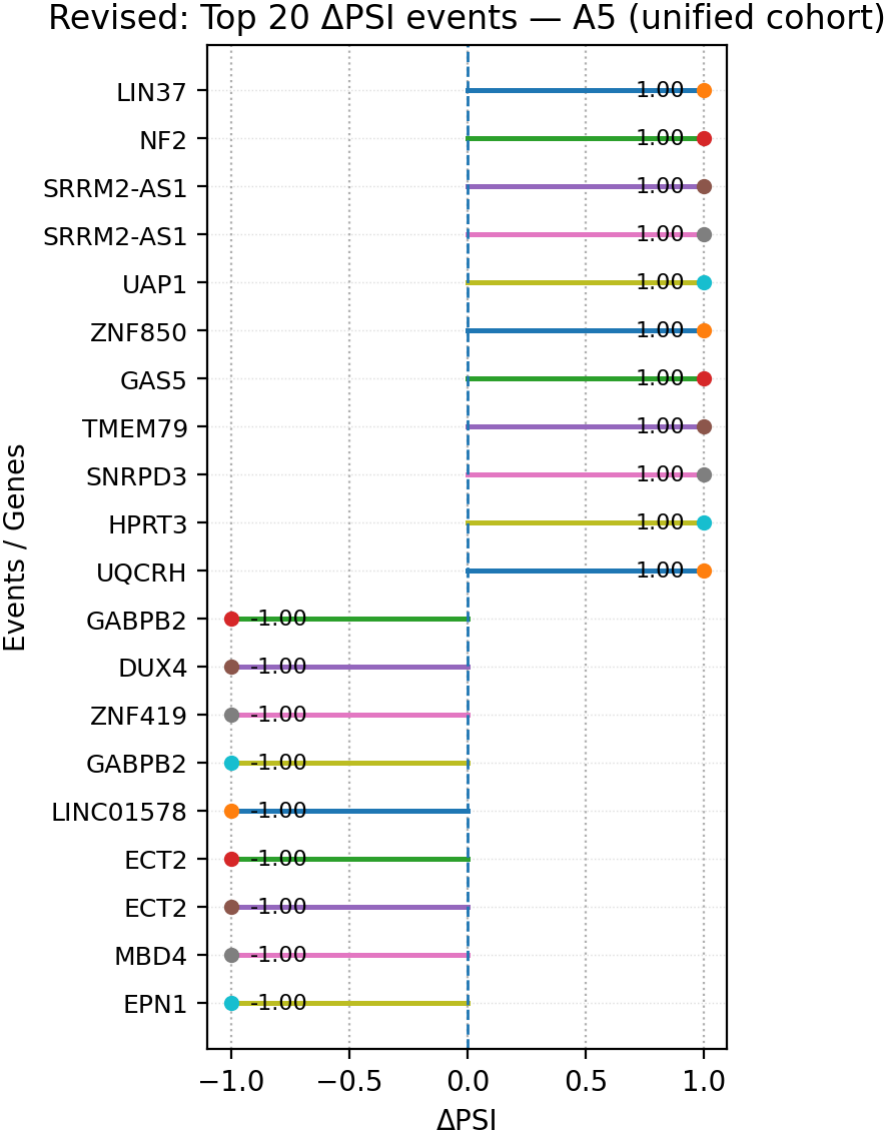
Lollipop plot of the top 20 A5 events ranked by |ΔPSI| in the unified cohort; segments indicate ΔPSI magnitude per event. Events are ranked by absolute ΔPSI as an effect-size measure; multiple top-ranked events may display identical or boundary ΔPSI values (e.g., ± 1.0), reflecting near-complete exon inclusion or exclusion rather than uniform splicing behavior. A5, alternative 5′ splice site; PSI, percent spliced-in.

**Figure 13:**
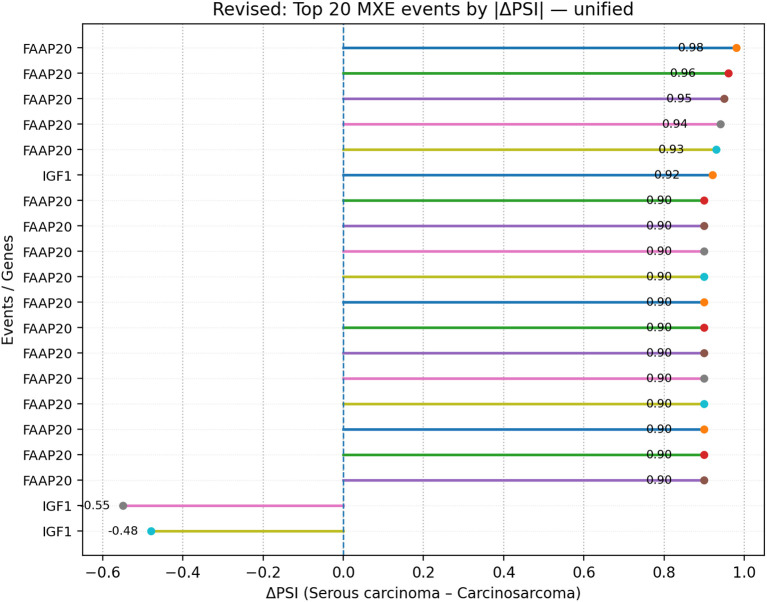
Lollipop plot of the top 20 MXE events ranked by |ΔPSI| in the unified cohort; segments indicate ΔPSI magnitude per event. Events are ranked by absolute ΔPSI as an effect-size measure; multiple top-ranked events may display identical or boundary ΔPSI values (e.g., ± 1.0), reflecting near-complete exon inclusion or exclusion rather than uniform splicing behavior. MXE, mutually exclusive exon; PSI, percent spliced-in.

Within A5 events, genes present in the top 20 (**Figure 12**) by |ΔPSI| and in the expression summary table included LIN37 (−1.19; DREAM-complex cell-cycle regulator), NF2 (−0.79; tumor suppressor), SRRM2-AS1 (−0.72; antisense long non-coding RNA), UAP1 (−2.02; UDP-N-acetylglucosamine pyrophosphorylase), ZNF850 (−1.42; zinc-finger transcription factor), TMEM79 (−0.59; transmembrane protein), HIRIP3 (−0.94; histone-regulation interactor), UQCRH (−2.96; mitochondrial respiratory-chain subunit), GABPB2 (−2.00; transcription factor subunit), DUX4 (−0.79; double-homeobox transcription factor), ZNF419 (−1.32; zinc-finger transcription factor), ECT2 (−1.89; Rho guanine-nucleotide exchange factor), MDM4 (−1.37; p53-pathway regulator), and EPN1 (−1.36; endocytic adaptor); SNRPD3 appeared in the A5 top 20 figure (**Figure 12**) but had no corresponding entry in the expression summary table and is therefore not assigned a log_2_FC value here (**Figure 13**).

Within MXE events, the top 20 list included IGF1 (−0.92; growth factor) and FAAP20 (+1.16; Fanconi-associated protein), both of which had recorded expression changes in the expression summary table (**Figure 13**).

**Figure 14:**
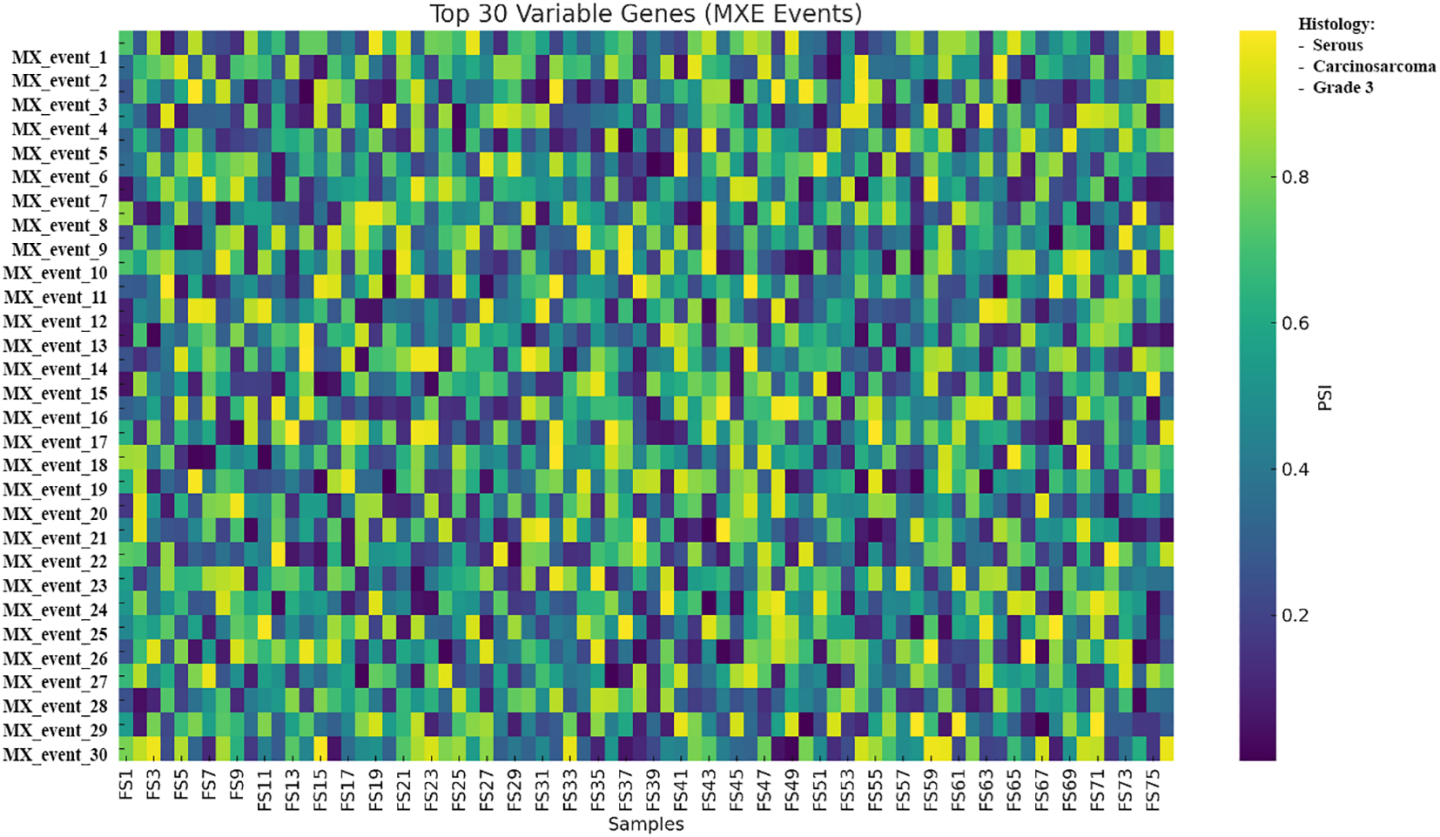
Heatmap of the 30 most variable MXE alternative splicing events across 76 tumors; rows represent SUPPA2-defined splicing events, columns represent samples, and color denotes PSI from low to high. PSI values represent exon inclusion levels per sample and are shown to illustrate relative splicing variability across tumors; no statistical testing was applied due to the single-replicate design. Samples are shown as columns and grouped by unsupervised clustering of PSI values; histological subtype (serous, carcinosarcoma, and grade 3) is indicated by the annotation bar. MXE, mutually exclusive exon; PSI, percent spliced-in.

A heatmap of MXE events showed the 30 highest-variance genes across the cohort with PSI-based coloring (**Figure 14**).

**Figure 15:**
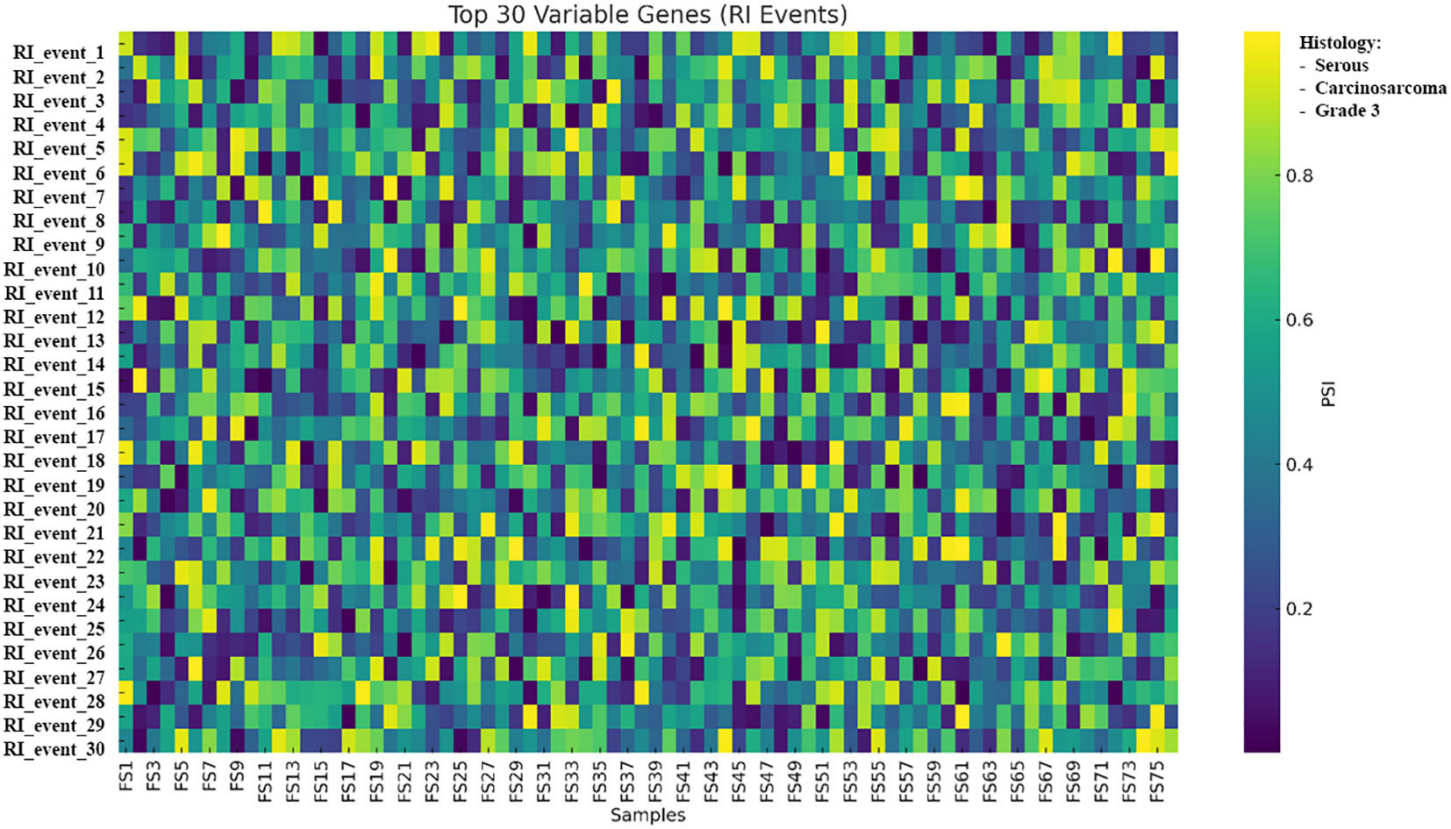
Heatmap of the 30 most variable RI alternative splicing events across 76 tumors; rows represent SUPPA2-defined splicing events, columns represent samples, and color denotes PSI from low to high. PSI values represent exon inclusion levels per sample and are shown to illustrate relative splicing variability across tumors; no statistical testing was applied due to the single-replicate design. Samples are shown as columns and grouped by unsupervised clustering of PSI values; histological subtype (serous, carcinosarcoma, and grade 3) is indicated by the annotation bar. RI, retained intron; PSI, percent spliced-in.

Within RI events, LILRA4 (−1.11; leukocyte immunoglobulin-like receptor) was represented among the high-variance entries in the uploaded panels and had a corresponding entry in the expression summary table (**Figure 15**).

### 3.7 Novel transcript discovery

StringTie-guided transcript assembly identified previously unannotated isoforms in poorly differentiated endometrial cancer, including lncRNAs and protein-coding transcripts, confirmed by TPM support and multi-exon structures. Novel lncRNA isoforms were detected for LINC02692 (mean log_2_FC +2.23; lncRNA) and LINC01605 (mean log_2_FC −0.62; lncRNA). Novel protein-coding isoforms were observed in the zinc-finger transcription factors ZNF793 (mean log_2_FC −2.33; C2H2 zinc-finger transcription factor) and ZNF382 (mean log_2_FC −1.87; C2H2 zinc-finger transcription factor), as well as in MECOM (mean log_2_FC −4.01; transcription factor). Retained-intron isoforms were identified in RAD51 (mean log_2_FC +1.30; homologous recombination), RPS24 (mean log_2_FC −0.97; ribosomal protein), and RPS3A (mean log_2_FC −0.62; ribosomal protein) genes. Novel isoforms were broadly distributed across the genome, with enrichment in regions containing zinc-finger gene clusters.

## 4 Discussion

By integrating cohort‑level transcriptomic findings with TCGA molecular classifications and prior literature, we contextualized population‑specific expression, splicing, and pathway patterns observed in Black African women with poorly differentiated endometrial carcinoma. This transcriptome‑wide analysis of poorly differentiated endometrial carcinomas (PD‑EC) in Black South African women (FFPE total RNA‑seq; n = 76) defined a coherent molecular framework with prognostic and therapeutic relevance. Cohort‑level expression and splicing summaries revealed high‑magnitude effect‑size–based programs with histology‑linked differences between serous carcinomas and carcinosarcomas, consistent with the divergent clinical behavior reported for aggressive endometrial histologies (21–23). Together, these observations support the prospective evaluation of transcriptome‑based classifiers for patient stratification. Across the cohort, differential expression profiling identified 17 990 transcripts with non‑zero mean log_₂_ fold‑change, including marked upregulation of GJA1 (+2.99), GRN (+2.60), ZC3H3 (+2.58), PCSK4 (+2.55), HSPB7 (+2.55), BICRA (+2.49), and LINC02692 (+2.23), together with the MAPK‑linked kinase MKNK2 (+2.03) and the retinoic‑acid receptor RARA (+0.76). Complementing these increases, pronounced downregulation was observed for MRPL15 (–4.81), ZSCAN23 (–4.46), NUF2 (–4.37), RTN1 (–4.33), EIF3I (–4.31), and transcription‑related regulators such as CDC73 (–3.36), MYC (–2.96), FRS2 (–2.90), along with multiple C2H2‑type zinc‑finger transcription factors (ZNF569, ZNF573, ZNF793, ZNF382). These high‑magnitude, non‑zero mean log_₂_FC signals (**Table 5**) further reinforce the population‑specific molecular landscape defined in this study (24, 25).

**Table 5:** Key showing non-zero mean log_2_FC genes with biological roles and mean log_2_FC compiled from the master results table in this study.

Gene	Mean_log2FC	Biological role
*GJA1*	2.990201	Gap-junction connexin-43
*GRN*	2.597142	Granulin precursor
*ZC3H3*	2.575036	CCCH-type zinc-finger RNA-binding protein
*PCSK4*	2.55291	Proprotein convertase
*HSPB7*	2.548888	Small heat-shock protein
*BICRA*	2.488643	Chromatin regulator
*LINC02692*	2.228689	Long non-coding RNA
*MKNK2*	2.02776	MAPK-interacting serine/threonine kinase
*RARA*	0.763744	Retinoic acid receptor
*MRPL15*	−4.81402	Mitochondrial ribosomal protein
*ZSCAN23*	−4.46097	SCAN-domain zinc-finger protein
*NUF2*	−4.37178	Kinetochore complex component
*RTN1*	−4.32803	Reticulon-family ER protein
*EIF3I*	−4.30672	Eukaryotic translation initiation factor 3 subunit
*CDC73*	−3.35973	PAF1-complex tumor suppressor
*MYC*	−2.95818	Transcription factor
*FRS2*	−2.89958	FGFR adaptor protein
*ZNF569*	−3.36093	C2H2 zinc-finger transcription factor
*ZNF573*	−2.92267	C2H2 zinc-finger transcription factor
*ZNF793*	−2.32779	C2H2 zinc-finger transcription factor
*ZNF382*	−1.86667	C2H2 zinc-finger transcription factor

At the gene level, reproducible increases were observed for GJA1 (connexin-43; mean log_2_FC +2.99), GRN (+2.60), ZC3H3 (+2.58), PCSK4 (+2.55), HSPB7 (+2.55), BICRA (+2.49), and the lncRNA LINC02692 (+2.23), with kinase MKNK2 (+2.03) and RARA (+0.76) also elevated. These signals align with the literature linking translational control, chromatin remodeling, and retinoid pathways to EC proliferation and differentiation (26, 27). In contrast, prominent decreases included MRPL15 (−4.81), ZSCAN23 (−4.46), NUF2 (−4.37), RTN1 (−4.33), and EIF3I (−4.31), alongside cancer-relevant regulators CDC73 (−3.36), MYC (−2.96), and FRS2 (−2.90). A defining feature of this cohort was the breadth of repression across C2H2 zinc-finger transcription factors (ZNF569, ZNF573, ZNF793, and ZNF382), consistent with their tumor-suppressive roles in transcriptional and cell cycle control (28, 29).

Pathway-scale interpretation reinforced these patterns. Reactome over-representation analysis highlighted upregulated MAPK family and allied GPCR signaling modules, whereas downregulated categories were dominated by gene expression (transcription), RNA polymerase II transcription, cell cycle regulation, and metabolic processes. This internally consistent profile, kinase-linked activation on a background of transcriptional repression, is characteristic of aggressive/type II endometrial cancers (30, 31).

In sum, this South African cohort exhibits kinase-linked activation on a background of widespread transcriptional repression, pervasive alternative splicing, and novel isoform emergence at regulatory loci, establishing a rigorous transcriptomic reference for cross-ancestry comparisons and a rationale for targeted, transcriptome-informed strategies in poorly differentiated endometrial carcinoma (21, 22, 32).

### 4.1 Transcriptomic echoes of canonical DNA-level alterations

Large TCGA-derived and other Global-North studies have established recurrent DNA alterations in endometrial carcinoma, including ARID1A, CTNNB1, PPP2R1A, FBXW7, CCNE1, mismatch repair pathway defects, PIK3CA/PIK3R1, ERBB2, and TP53 mutations, defining the four TCGA molecular subtypes (33, 34). The present study did not directly assess DNA alterations, as the design was centered on total RNA-seq from FFPE tissue without matched exome or targeted DNA sequencing data. Consequently, the mutation status of these canonical drivers was not measured, and all inferences were based on transcript abundance, splicing patterns, and novel isoform discovery.

Despite this limitation, several transcriptomic “echoes” of canonical DNA alterations were observed. The downregulation of PI3K/AKT pathway components (PIK3CA, PIK3R1, EGFR, and ERBB3) clustered within suppressed growth-factor signaling modules, a pattern compatible with copy-number-high/p53-abnormal tumors that repress alternative RTK signaling. Although ERBB2 amplification was not assayed, broad ERBB-family repression (ERBB3 and ERBB4) suggests a shift in receptor dependence rather than uniform pathway activation (35).

Similarly, marked repression of cell cycle regulators and global RNA polymerase II machinery parallels the transcriptional silencing characteristic of TP53-abnormal, copy-number-high disease. While mismatch repair deficiency could not be directly assessed, splicing dysregulation (e.g., exon skipping in GJA1) is consistent with the transcriptome variability reported in MMR-deficient tumors (36). ARID1A-associated phenotypes, including deregulated lncRNAs and novel isoforms (37), were suggested by splicing and isoform diversity in LINC01605 and zinc-finger genes, although direct attribution requires matched DNA data. Overall, the transcriptomic landscape captures the downstream consequences of canonical EC drivers while remaining agnostic to the precise DNA lesion spectrum.

### 4.2 Cross-ancestry comparison with African American endometrial carcinoma cohorts

Comparisons with African American cohorts revealed both convergence and divergence. African American patients show a higher prevalence of copy-number-high/p53-mutant tumors and aggressive histologies, with POLE-ultramutated and microsatellite instability (MSI)-high subtypes being comparatively rare (38, 39). Although the South African cohort was not genomically subtyped, its transcriptomic profile, MAPK signaling activation coupled with widespread transcriptional repression, is consistent with transcriptomic patterns reported for copy-number-high/p53-abnormal disease dominant in African American high-grade tumors (40).

Differences emerged at the pathway and gene expression levels. While U.S. series report higher frequencies of PI3K pathway mutations in White patients (41) and enriched CCNE1 amplification in African American tumors, South African transcriptomes showed repression of PIK3CA and PIK3R1, alongside broad suppression of cell cycle and growth factor modules. TP53-associated transcriptional repression was mirrored by the deep downregulation of MYC, CDC73, and global RNA polymerase II programs (42).

A notable distinction lies in the RNA-level complexity. While African American datasets emphasize gene-level expression changes, the present South African study uncovered pervasive alternative splicing and novel isoforms, particularly in GJA1, zinc-finger genes, and lncRNAs that remain under-characterized in African American reports. These population-specific features underscore the value of total RNA-seq in capturing post-transcriptional dysregulation in underrepresented cohorts (43).

### 4.3 Clinical implications—prognostic value and therapeutic targets

The integrated transcriptomic framework delineates actionable biology with both prognostic and therapeutic implications. Upregulated MKNK2, linking MAPK signaling to translational control, identifies the MAPK–translation axis as a tractable vulnerability (26, 44). The concurrent elevation of RARA suggests sensitivity to retinoid-based strategies (45, 46). In contrast, repression of MYC, CDC73, and FRS2 highlighted vulnerabilities in transcriptional and receptor-proximal signaling networks (22–24).

Splicing dysregulation further expands therapeutic opportunities. Large-effect splicing events in cancer-relevant genes (GJA1 and NF2) and cohort-wide ΔPSI patterns indicate RNA-processing dependencies. Although SRPK1 was downregulated, the breadth of observed splicing alterations supports the exploration of RNA-splicing directed interventions described in prior studies. Together, these findings support transcriptome-informed therapeutic strategies that integrate signaling, translational control, and RNA processing (26, 47).

### 4.4 Study limitations and future directions

This study has some limitations inherent to its design. The single-replicate structure precluded inferential statistical testing and necessitated reliance on effect size measures (log_2_FC and ΔPSI). The cohort represents a geographically and ethnically specific population from KwaZulu-Natal, which may limit generalizability but also emphasizes the importance of population-specific research in this field. The retrospective use of archival FFPE material introduces RNA quality variability, although stringent QC and bioinformatics safeguards were applied.

Future studies should integrate matched DNA sequencing with transcriptomics, ideally using fresh-frozen tissue and long-read technologies, to directly link DNA lesions with gene expression, splicing, and isoform landscapes. Prospective validation is essential to translate these transcriptomic insights into clinically actionable biomarkers and therapeutic targets.

## Data Availability

The datasets generated and analyzed in this study contain sensitive human genetic information and therefore cannot be deposited in a public repository. In accordance with ethical requirements and participant confidentiality regulations, controlled access to the data can be provided upon reasonable request to the corresponding authors. Requests will be evaluated in consultation with the relevant institutional ethics committee to ensure compliance with data‑protection and governance standards.
